
JOURNAL INFORMATION
==============================
NLM Title Abbreviation: JBMR Plus
Journal ID: JBMR Plus
NLM Journal ID: 101707013
Journal ID: jbmrplus

JBMR Plus

EISSN: 2473-4039
Publisher: Oxford University Press

ARTICLE INFORMATION
==============================
PMCID: PMC13503960
DOI: 10.1093/jbmrpl/ziag134
Article ID: ziag134
Article version: 1
Subjects: Abstracts, AcademicSubjects/MED00010, AcademicSubjects/MED00160, AcademicSubjects/MED00250

10th International Conference on Osteoimmunology: Interactions of the Immune and Skeletal Systems

Publication date: 2026 Sep
Electronic publication date: 2026 Aug 25
Volume: 10
Issue: Suppl 4
First page: iv1
Last page: iv61
Copyright: © The Author(s) 2026. Published by Oxford University Press on behalf of the American Society for Bone and Mineral Research.
Copyright year: 2026
License: This is an Open Access article distributed under the terms of the Creative Commons Attribution License (https://creativecommons.org/licenses/by/4.0/), which permits unrestricted reuse, distribution, and reproduction in any medium, provided the original work is properly cited.
License URL: https://creativecommons.org/licenses/by/4.0/

Page count: 1

==============================

### Conference Chairs

**Iannis Adamopoulos**
Harvard University
Boston, Massachusetts, United States

**Yongwon Choi**
University of Pennsylvania School of Medicine
Department of Pathology and Laboratory Medicine
Philadelphia, Pennsylvania, United States

**Lionel Ivashkiv**
Hospital for Special Surgery
New York, New York, United States

**Mary Nakamura**
University of California, San Francisco
Arthritis/Immunology Section (111R)
San Francisco, California, United States

**Georg Schett**
Uniklinikum Erlangen
Department of Medicine 3 – Rheumatology and Immunology
Erlangen, Baden-Wuerttemberg, Germany

**Natalie Sims**
The University of Melbourne
Department of Medicine at St. Vincent's Hospital
Fitzroy, Australian Capital Territory, Australia

**Hiroshi Takayanagi**
The University of Tokyo, Graduate School of Medicine and Faculty of Medicine
Department of Immunology
Tokyo, Tokyo, Japan

**Deborah Veis**
Washington University in St. Louis
School of Medicine
St. Louis, Missouri, United States

### Meeting Summary

Hiroshi Takayanagi, Mary Nakamura, Iannis E Adamopoulos and Deborah Veis

**Day 1: Stroma, Stem and Progenitor Cells: New Frontiers at the Bone, Immune and Vascular Interface**

The first day of the conference chaired by Iannis Adamopoulos and Hiroshi Takayanagi highlighted how rapidly the field of osteoimmunology is expanding beyond classical bone resorption and formation, toward a more integrated view of bone as a dynamic immune, vascular, metabolic and stem cell organ. Across Sessions I and II, speakers explored how specialized niches in bone marrow, growth plate, vasculature and stromal compartments shape skeletal homeostasis, inflammation, regeneration and disease.

The session opened with Noriaki Ono who presented new insights into the endosteal niche at the crossroads of bone and immune cells. Building on the concept that PTHrP positive resting zone chondrocytes serve as skeletal stem cells in the postnatal growth plate, his talk emphasized how PTHrP controls the orderly transition of chondrocyte differentiation and prevents ectopic hypertrophic conversion through GDF5 pathways.

Andrei Chagin further expanded this theme by describing a dual stem cell organization in growth plates. His presentation distinguished two resting zone stem cell subsets, including a Wnt enriched, highly stem like GP1 population and a GP2 population marked by different molecular features. This concept provides a new framework for understanding how the growth plate sustains longitudinal bone growth while balancing stemness, proliferation and hypertrophic differentiation.

Anjali Kusumbe’s lecture focused on skeletal vasculature and vascular niches, emphasizing endothelial diversity across space, time and function. Her work has been central to defining specialized bone vessels as niche structures that regulate bone formation, hematopoiesis and tissue aging. Earlier studies showed that age associated decline of niche forming skeletal vessels can impair bone and hematopoietic environments, while more recent work continues to reveal site specific vascular and stromal heterogeneity in skull, vertebral and long bone marrow niches.

The second session shifted toward inflammatory and regenerative mechanisms in stromal, stem and progenitor cell biology. Masayuki Tsukasaki presented a vascular derived RANKL and OPG axis controlling arterial calcification independently of the skeleton, illustrating how classical bone regulatory molecules can act in vascular disease. Ritu Chakravarti introduced multilevel regulation of osteoclastogenesis by 14-3-3ζ at the inflammation and bone interface.

Several talks emphasized bone as an active systemic responder to inflammation. Stavroula Kousteni described bone-derived inflammatory SAA1 as a regulator of stem cell state and persistence in myeloid malignancies. Yasuhiro Kobayashi addressed inflammaging, focusing on endothelial RANK signaling.

A recurring theme was the intimate connection between immune cells and skeletal repair. Salwa Suliman and colleagues highlighted regulatory T cells as orchestrators of mesenchymal stromal cell mediated bone regeneration. Kazutaka Nakamura and colleagues described a stromal niche supporting osteoclastogenesis in giant cell tumor of bone. Daniela Paternina Martinez showed that IL 17RA blockade can uncover alternative osteogenic pathways driven by IL 17F through BMP and Wnt signaling. Jessica De Angelis presented evidence that bone can rapidly sense inflammation and secrete Lipocalin 2, reinforcing the concept that skeletal tissue participates actively in immune communication.

Together, the session portrayed bone as a highly organized, immune responsive and metabolically adaptable organ. Stem cells, stromal cells, endothelial cells, immune cells and osteoclasts do not act in isolation. They form interconnected niches that determine skeletal growth, inflammatory bone loss, regeneration, vascular calcification, aging and malignancy. This integrated view captures the current direction of osteoimmunology: from bone and immune crosstalk toward a broader biology of tissue niches, systemic inflammation and organ level adaptation.

**Day 2: New insights in Osteoclast biology, Inflammation and Bone**

Day 2, chaired by Gerhard Krönke and Dirk Elewaut, began with a session focused entirely on osteoclasts. Yuji Mishina discussed modes by which osteoclasts control osteoblasts, including direct contact that leads to osteoblast cell death in vivo. He showed data indicating that this communication is largely by transfer of specific proteins, suggesting that interruption of this mechanism might preserve bone mass.

Next, we were introduced to the role of super-enhancer derived RNAs in osteoclasts by Kyung Park-Min. Her group used human osteoclast cultures to find osteoclast-specific actively produced transcripts, and showed that some of these non-coding RNAs control originating from the NFATc1 and MYC super-enhancers control osteoclastogenesis. The next step will be to determine if this new mode of regulation is altered in disease states such as rheumatoid arthritis.

Aline Bozec delved into metabolism by osteoclasts, describing roles for L-arginine and L-ornithine in inflammatory bone loss. Interestingly, she showed that L-ornithine increases synthesis of the neurotransmitter GABA, which acts via binding to β-catenin, to decrease osteoclastogenesis. Fanxin Long also delved into the bioenergetics of osteoclasts, focusing on the glucose transporter Glut1 and fatty acid oxidation. His work follows previous studies showing significant sex differences in the mouse models that remain incompletely explained.

We learned about lysosomal transporters in osteoclasts from Nathan Pavlos. These organelles are critical to the bone resorption by osteoclasts, but not all highly expressed transporters control this function.

Ara Nazarian took a completely different approach, describing an integrated computational model coupling mechanical regulation with biological healing. He noted that most models skip the soft callus phase when important inflammation occurs and emphasized the importance of “ground truth” experimental data.

Gabriel Mbalaviele took us deeper into the mechanisms of inflammation and regulation of bone mass, focusing on pyroptosis, an inflammatory cell death. He first described the surprising result that low-dose LPS stimulates periosteal, but not endosteal, bone formation by causing cell death in the bone marrow, dependent on GSDMD. He next showed that deficiency in GSDME, a mediator of inflammasome-independent pyroptosis, leads to age-associated bone loss. The take-home message is that pyroptosis can have either beneficial or harmful effects on bone, depending on the gasdermin and biological context.

The day ended with short talks by Carina Scholtysek and Sanya Novak. Carina continued the metabolism theme, discussing the role of Nrf1 in driving osteoclast differentiation using conditional knockout mice. Sanya added to the discussion of inflammation by describing a novel CD115+Adgre+ osteoclast progenitor population.

**Day 3: Advances in Arthritis**

Sessions on the third day chaired by Mary Nakamura and Masayuki Tsukasaki were focused on osteoimmune roles in inflammatory and degenerative arthritis along with talks demonstrating critical interactions between the central and peripheral nervous system and bone.

The session opened with an exciting discussion by Aristotelis Tsirigos regarding use of artificial intelligence with imaging and -omics technologies to decode the microenvironment and gene regulation to advance studies in precision medicine. He showed how models are being built to construct virtual cells to simulate, predict, and steer cell behavior, with the eventual goal of predicting patient specific drug-target interactions. While studies have primary focused to date on cancer, it is clear that these types of studies will be applicable to bone and autoimmune disease.

Several talks discussed the role of immune signals in mouse models of arthritis. Yousef Abu-Amer discussed how IκBζ, an atypical member of the IκB protein family can act as inflammatory switch in myeloid cells to trigger inflammatory arthritis, with deletion improving arthritis in a serum transfer model. Gabriela Loots discussed immune cell influences in a post-traumatic arthritis mouse model with evidence suggesting a protective role for TREM2+ macrophages. Her findings suggest that novel immune targets may be useful as an intervention at the onset of injury to prevent development of post-traumatic osteoarthritis. Chris Ritchlin showed how a new humanized mouse model utilizing peripheral blood samples from patients was able to phenocopy psoriatic arthritis with transfer of cells and plasma into NSG mice. In a short abstract presentations, Vikrant Piprode discussed how progranulin promotes NLRP3 inflammasome mediated IL1β production in an inflammatory arthritis model and Georgiana Neag showed how signals from stromal cells interact with myeloid cells and osteocyte survival to form regulatory circuits that impact bone loss and inflammatory arthritis.

Expanding from immune-bone interactions, several talks focused on the role of the central and peripheral nervous system in regulating bone disease. Jason Ivanusic showed us beautiful visualization and 3D mapping of different types of nerves in and around the bone. Tetsuo Hasegawa showed us refined 3D imaging of whole mount synovium and discussed how cells from the dorsal root ganglia communicate with synovial macrophages in inflammatory arthritis. Mone Zaidi showed us how hypothalamic-produced FSH likely plays a major regulatory role in postmenopausal osteoporosis and demonstrated how a promising therapeutic, an FSH blocking antibody, is under development for treatment of osteoporosis, obesity and Alzheimer's disease. Dirk Elewaut showed us how GDF-15 is an important player mediating a brain-bone axis that controls inflammation and bone loss. In the final talk of the session, Gerhard Kröenke discussed how molecular profiling of macrophage-fibroblast crosstalk defines checkpoints orchestrating onset and resolution of inflammation. Synovial fibroblasts dominated intercellular signaling as a source of cytokines, chemokines and growth factors at the onset of arthritis in the serum transfer model during arthritis onset, leading to the recruitment of pro-inflammatory *Il1b*$^{+}$ macrophages that eventually differentiated into *Spp1*$^{+}$ anti-inflammatory macrophages. The expression of osteopontin by these cells was of importance to downregulate synovial inflammation.

Overall, the session highlighted the importance of interacting signals between multiple cell types that integrate the nervous system, stromal cells, immune cells and bone in both maintaining homeostasis and promoting pathological states in the bone and joints.

**Day 4: Infection and Cancer in Bone**

The final day of talks was chaired by Deborah Veis and Aline Bozec. The first part of the day focused on bone infection. Gowri Muthukrishnan discussed mechanisms facilitating Staphylococcal infection leading to osteomyelitis, using humanized mouse models and patient samples to highlight remodeling of the T cell compartment and increased expression of checkpoint proteins. Janko Nikolich discussed how age-related immune changes play a significant role in propagation of alphavirus mediated arthritis, focusing on Chikingunya which can lead to a chronic inflammatory arthritis more commonly in older individuals related to changes in their immune response via dysregulated TGFβ. Gulnz Guliyeva gave a short talk on her work focused on the innate immune receptor Dectin 1, showing a significant role for this receptor in the development of periodontal bone loss.

Focusing on cancer, Hiroshi Takayanagi discussed how specific macrophage subsets that play a role in tissue remodeling contribute to the development of bone metastases. Dorothy Sipkins gave an interesting talk highlighting the role of bone in central nervous system metastases, demonstrating how breast cancer cells that expressed the cell surface receptor integrin α6 were able to spread along the outer surface of blood vessels by migration through calvarial bone marrow and exiting the bone marrow through channels in the calvarial bone to the leptomeninges. Serk In Park discussed the role of B7H3 driven macrophage regulation in skeletal niche of bone metastases and Betsy Young showed evidence in animal models that STING activation reshapes the tumor microenvironment and can lead to tumor regression in osteosarcoma.
Additional talks included Eddie Schwarz discussing the novel role of telocytes and mast cells in the synovial lymphatic system and inflammatory arthritis. He explained that telocytes are stromal cells around lymphatics that support tissue organization and cell communication which are reduced in RA. Prodromos Sidiropoulos discussed evaluation of peripheral blood gene expression signatures in a unique early inflammatory arthritis cohort and described association of gene expression with long term outcomes. An interesting talk by Hiroshi Asahara described gene mutations identified in elite athletes and results of placing these gene mutations in mice. His talk highlighted the importance of tendons in the resulting athletic giftedness. Takahiro Morita expanded on this theme describing the mechanical regulation of structural and metabolic remodeling in tendon during aging and environmental adaption. In additional short talks, Xin Tian showed us his study of subchondral bone formation in OA that was surprisingly driven primarily by changes in osteogenesis without evidence of increased bone resorption or sclerostin down regulation. Christiane Schirm told us about a novel *in vitro* method to generate and study osteomorphs, cells derived from osteoclast fission, which should enable additional studies to better understand their function and roles in diseases of bone loss.

In addition to the oral presentations, we held engaging poster sessions and had many discussions over dinners at local restaurants, sampling the local delicacies. At the farewell dinner, 14 trainee travel awards were granted. The recipients were Jessica DeAngelis, Gulnaz Guliyeva, Marina Kleim, Parker Kooima, Heidi Lunze, Takahiro Morita, Kazutaka Nakamura, Vikrant Piprode, Hideaki Sabe, Christiane Schorm, Xin Tian, Renee van der Cruyssen, Elizabeth Young, and Tonxin Zhu. We also recognized our sponsors, the American Society for Bone and Mineral Research, the Japanese Society for Bone and Mineral Research, and Regeneron, who helped us provide so much trainee support. The 10$^{th}$ international meeting also marked the retirement of Mary Nakamura from the organizing committee after 20 years of service. A farewell award was given to Dr. Nakamura to congratulate her and extend our gratitude for her inspirational service to young trainees all over the world and her long-standing efforts promoting the field of osteoimmunology. We are looking forward to welcoming you again at the next Osteoimmunology meeting which will take place in 2028!

### Table of Contents

Conference Chairs ........ 1

Meeting Summary ........ 3

Abstracts ........ 10

ENDOSTEAL NICHE AT THE CROSSROAD OF BONE AND IMMUNE CELLS ........ 10

A VASCULAR-DERIVED RANKL-OPG AXIS DETERMINES ARTERIAL CALCIFICATION INDEPENDENTLY OF THE SKELETON ........ 11

BLOCKADE OF IL-17RA HIGHLIGHTS ALTERNATIVE OSTEOGENIC PATHWAYS DRIVEN BY IL-17F VIA BMP/WNT SIGNALLING ........ 12

NEW MODES FOR SPATIO-TEMPORAL COUPLING OF BONE FORMATION AND BONE RESORPTION GOVERNED BY OSTEOCLASTS ........ 13

THE FINGERPRINT OF BONE RESORPTION: DEFINING THE OSTEOCLAST SPECIFIC SIGNATURE ........ 14

LYSOSOMAL TRANSPORTERS IN BONE HEALTH & DISEASE: THE INS AND OUTS OF OSTEOCLASTS ........ 15

SPATIO-FUNCTIONAL HETEROGENEITY OF RESIDENT MACROPHAGES FORMING TISSUE- SPECIFIC CHRONIC INFLAMMATION ........ 16

DYSREGULATED CCL CHEMOKINE SIGNALING AS A SPATIOTEMPORAL DRIVER OF FRACTURE NON-UNION IN HIGH-RISK POPULATIONS: A MULTI-OMICS AND GENOMECHANICAL FRAMEWORK ........ 17

HOW INFLAMMASOME-DEPENDENT AND -INDEPENDENT PYROPTOSIS SHAPE BONE PATHOPHYSIOLOGY ........ 18

MITOCHONDRIAL COMMANDERS: THE NUCLEAR RESPIRATORY FACTOR 1 (NRF1) DRIVES OSTEOCLAST DIFFERENTIATION AND SKELETAL BALANCE ........ 19

FROM IMAGES TO OMICS: AI-DRIVEN TECHNOLOGIES FOR DECODING THE IMMUNE MICROENVIRONMENT AND GENE REGULATION ........ 21

PROGRANULIN PROMOTES NLRP3 INFLAMMASOME-MEDIATED IL-1B PRODUCTION AND SUBSEQUENT INFLAMMATORY BONE LOSS ........ 22

3D IMAGING OF THE SYNOVIUM DEFINES AN IMMUNOLOGICAL DEFENCE SYSTEM AT THE BLOOD-JOINT BARRIER ........ 23

OSTEOIMMUNOLOGY OF POST-TRAUMATIC OSTEOARTHRITIS IN MICE ........ 24

THE ROLE OF IKBZ AS AN INFLAMMATORY SWITCH IN ARTHRITIS ........ 25

AXIAL PSORIATIC ARTHRITIS IS PHENOCOPIED IN A HUMANIZED MOUSE MODEL .. 26

OSTEOIMMUNOLOGY OF BONE INFECTIONS ........ 27

NOVEL ROLES OF TELOCYTES AND MAST CELLS WITHIN THE SYNOVIAL LYMPHATIC SYSTEM, JOINT HOMEOSTASIS, AND INFLAMMATORY-EROSIVE ARTHRITIS ........ 28

AGE-RELATED MECHANISMS OF ALPHAVIRUS-MEDIATED ARTHRITIS – LESSONS FOR TISSUE REMODELING IN OLDER AGE ........ 29

THE CONTRIBUTION OF DECTIN-1 TO PERIODONTAL BONE LOSS .................................. 30
LOOKING THROUGH A DEVELOPMENTAL LENS TO BETTER UNDERSTAND THE OSTEOSARCOMA TUMOR MICROENVIRONMENT ................................................................ 31
MECHANICAL REGULATION OF STRUCTURAL AND METABOLIC REMODELING IN TENDON DURING AGING AND ENVIRONMENTAL ADAPTATION .................................... 32
ESTABLISHING AN IN VITRO APPROACH TO GENERATE OSTEOMORPHS ..................... 33
CD14ST AND OSTEOIMMUNOLOGICAL BIOMARKERS : EFFECTS OF COVID19 ON BONE REMODELING ........................................................................................................ 34
A BIOPRINTED JOINT-ON-A-CHIP PLATFORM FOR MODELLING OSTEOIMMUNE INTERACTIONS IN INFLAMMATORY ARTHRITIS ................................................................ 35
ALTERING HOST MICROENVIRONMENT IS CRITICAL FOR SUCCESS OF CELL THERAPY FOR OSTEOGENESIS IMPERFECTA ........................................................................... 36
ANNEXINA1-DECTIN 1 AXIS IS A KEY REGULATOR OF OSTEOCLASTOGENESIS UNDERLYING IRRADIATION INDUCED BONE LOSS ........................................................... 37
ANTIRESORPTIVE THERAPY REWIRES OSTEOIMMUNE SIGNALING IN MRONJ: THE ROLE OF ANTIMICROBIAL PEPTIDES IN BONE TURNOVER IMBALANCE ...................... 38
BRIDGING IMMUNITY AND BONE: DECTIN-1–RANK SIGNALING REGULATES HUMAN OSTEOCLAST DIFFERENTIATION ............................................................................ 39
CHARACTERIZATION OF A PRO-RESOLVING EOSINOPHIL SUBSET IN RHEUMATOID ARTHRITIS AND ITS REGULATION BY ANTI-RHEUMATIC TREATMENT ........................................................................................................................................ 40
CSF1R⁻ MACROPHAGES GOVERN THE SECOND WAVE OF NEUTROPHILS FOR BONE REPAIR ...................................................................................................................... 41
DECHIPERING THE ROLE OF P4HB IN OSTEOCLASTS AND IN IN VIVO BONE HOMEOSTASIS ......................................................................................................... 42
DIRECT AND OSTEOBLAST-MEDIATED EFFECTS OF IL-1B, TNF-A AND IFNΓ ON HUMAN OSTEOCLASTOGENESIS ............................................................................. 43
DKK1 INHIBITION ALLEVIATES MITOCHONDRIAL DYSFUNCTION AND CELLULAR SENESCENCE VIA THE CGAS-STING-IFN-BETA-STAT3 PATHWAY IN AGED BONE ..... 44
DONOR MICROBIOTA AGE DELAYS FRACTURE PAIN RESOLUTION AND CALLUS MINERALIZATION IN FEMALE MICE ...................................................................... 45
EARLY HEMATOPOIESIS IN THORACIC AND ABDOMINAL AORTIC ANEURYSMS ...... 46
GENETICALLY MODIFIED HOXB8 PROGENITOR CELLS FOR FUNCTIONAL ANALYSIS OF OSTEOCLASTOGENESIS ........................................................................................ 47
IMMUNE “TRAINING” DEMONSTRATED BY EXACERBATION OF INFLAMMATORY ARTHRITIS IS MEDIATED BY LYMPHOCYTES .................................... 48
INFLAMMATORY MEDIATORS AND MIGRATORY POTENTIAL OF OSTEOCLAST PROGENITORS IN TYPE 1 DIABETES MELLITUS ................................................................ 49
INVESTIGATING THE ROLE OF BISPHOSPHONATES ON IMMUNE CELLS FOR THERAPEUTIC DEVELOPMENT FOR OSTEONECROSIS OF THE JAW (ONJ) .................... 50
LOSS OF TFR2 UNLEASHES NEUTROPHIL-LIKE INFLAMMATORY PROGRAMMING IN OSTEOCLAST PROGENITORS AND DRIVES INFLAMMATORY BONE LOSS .................... 51
OSTEOSARCOMA HIJACKS LOCAL BONE MARROW HEMATOPOIESIS TOWARD MYELOID CELL EXPANSION AND ATF3 ACTIVATION ....................................................... 52
SIX HOUR DECALCIFICATION: A MICROWAVE ENHANCED WORKFLOW FOR 3D IMAGING OF MINERALIZED TISSUES ........................................................................ 53

SPLICING FACTOR SRSF1 CONTROLS SYSTEMIC INFLAMMATION METABOLISM AND FUNCTION OF MYELOID DERIVED SUPPRESSOR CELLS .................................................. 54

TARGETED ACTIVATION OF STAT3 IN BONE ENHANCES FRACTURE HEALING .......... 55

THE IMPACT OF CHRONIC COLITIS ON BONE MARROW NICHE STRUCTURE ............... 56

THREE-DIMENSIONAL VISUALIZATION OF COMPLETE VASCULAR NETWORK AND NEUTROPHIL CLUSTERS IN MURINE KNEE JOINTS DURING INFLAMMATORY ARTHRITIS .......................................................................................................................... 57

TIME-DEPENDENT CHANGES IN THE IMMUNE AND SKELETAL SYSTEMS FOLLOWING IRRADIATION AND HEMATOPOIETIC STEM CELL TRANSPLANTATION 58

TIME-RESTRICTED FEEDING ENGAGES THE GUT-IMMUNE-BONE AXIS TO ATTENUATE AGE-RELATED BONE LOSS ............................................................................. 59

**ENDOSTEAL NICHE AT THE CROSSROAD OF BONE AND IMMUNE CELLS**

Noriaki Ono
*School of Dentistry, UT Health Houston, Houston, United States*

The bone marrow endosteal niche lies at the interface between the skeletal and immune systems, providing a specialized microenvironment that supports both skeletal and hematopoietic stem and progenitor cells. We recently identified a population of Fgfr3-expressing endosteal stem cells that are abundant in young bone marrow and exhibit robust osteogenic potential. Building on this discovery, we identified new cell surface markers using antibody screening and are defining their dynamic interactions with hematopoietic cells within the endosteum. In parallel, we have investigated the developmental origins of the endosteal niche using morphology-guided cellular spatial transcriptomics (MCST), a high-resolution spatial transcriptomics platform developed in collaboration with computational biologists. By integrating MCST with advanced computational frameworks, including CytoSignal and TopoVelo, we reconstructed spatial cell-state transitions and intercellular signaling networks during endochondral bone development. These analyses uncovered a previously unrecognized orthogonal PTHrP-GDF5 signaling axis that coordinates chondrocyte hypertrophy and establishment of the endosteal niche. Together, these studies provide new insights into how developmental programs shape the bone marrow microenvironment and establish the cellular and molecular framework that governs lifelong interactions between skeletal and immune cells.

**A VASCULAR-DERIVED RANKL-OPG AXIS DETERMINES ARTERIAL CALCIFICATION INDEPENDENTLY OF THE SKELETON**

Masayuki Tsukasaki

*Department of Biochemistry, Showa Medical University, Tokyo, Japan*

Vascular calcification elevates the risk of cardiovascular events and mortality. The activity of osteoclast differentiation factor RANKL is fine-tuned by its decoy receptor osteoprotegerin (OPG). OPG deficiency results in both bone loss and vascular calcification, which has traditionally been attributed to the "calcium-shift" from the skeleton to the vasculature-a hypothesis that has never been validated in vivo. Using cell-type specific OPG conditional knockout mice, here we show that vascular smooth muscle cells (VSMCs) but not osteoblasts are the crucial source of OPG in the regulation of arterial calcification, underscoring the importance of the extra-skeletal function of RANKL. Combined deficiency of RANK indicates that RANK expressed by VSMCs drives vascular calcification. Anti-RANKL antibody more effectively inhibited the progression of vascular calcification than bisphosphonate in patients with osteoporosis. This study defines the vascular RANKL/OPG axis as a potential therapeutic target for the prevention of arterial calcification.

**BLOCKADE OF IL-17RA HIGHLIGHTS ALTERNATIVE OSTEOGENIC PATHWAYS DRIVEN BY IL-17F VIA BMP/WNT SIGNALLING**

Daniela Paternina Martinez[1], Sarah Brown[1], Mittal Shah[2], and Scott Roberts[1]

[1]*Comparative Biomedical Sciences, The Royal Veterinary College, London, United Kingdom*

[2]*Division of Surgery and Interventional Science, University College London, London, United Kingdom*

Radiographic axial spondyloarthritis (r-axSpA, also known as ankylosing spondylitis) is the most characterized spondyloarthritis regarding pathological bone formation (PBF), with TNF-α and IL-17 signaling implicated in sacroiliac or spine ankylosis. PBF is intimately associated with the periosteum, a highly vascularised membrane that covers all bones and mediates fracture repair. We have previously reported that IL-17A and F promote osteogenic differentiation (OD) and in vitro bone formation/mineralisation of human periosteum derived cells (hPDCs). IL-17F is a homologue of IL-17A, and binds to the same IL-17RA/IL-17RC complex to induce signalling. Herein, using an in vitro biomimetic assay, IL-17A induced a dose response effect on hPDC mineralisation at a range of 1-20ng/ml (8-16-fold change ($p \leq 0.01$) vs. control), whereas IL-17F showed no dose-associated trend (10-12-fold change ($p \leq 0.01$)) across the same range. Moreover, TNFα (500 pg/ml) exerted a synergistic effect on OD with both IL-17A and F, compared to either cytokine alone ($p \leq 0.01$). However, gene expression of key osteogenic markers (such as RUNX2 - master regulator of osteogenesis) were abrogated at 10ng/ml, indicating concentration-dependency. Interestingly, IL-17RA blockade with brodalumab decreased IL-17A-mediated OD to baseline ($p \leq 0.001$), but only partially reduced that of IL-17F ($p \leq 0.001$). RNAseq and STRING analysis revealed that IL-17A upregulated genes associated with osteoclast and Th17 differentiation, while IL-17F preferentially upregulated genes linked to osteogenesis (e.g. BMP2). Furthermore, IL-17RA blockade led to downregulation of genes associated with the TNFα/IL-6 signalling pathway with IL-17A. Interestingly, inhibition of IL-6R with tocilizumab reduced both IL-17F and IL-17A induced OD. IL-17F in the presence of brodalumab also regulated a BMP/Wnt signature consisting of genes such as WNT9A, SFRP1 and TMEM119, suggesting an IL-17RA independent mechanism of IL-17F mediated bone formation. These data support the hypothesis that IL-17F mediates PBF via alternate pathways to IL-17A, potentially through BMP/Wnt signalling. This is corroborated by previous reports that IL-17F (but not IL-17A) upregulates RUNX2, however further research is required to investigate the clinical relevance of these findings.

**NEW MODES FOR SPATIO-TEMPORAL COUPLING OF BONE FORMATION AND BONE RESORPTION GOVERNED BY OSTEOCLASTS**

Yuji Mishina[1], Maiko Sugihara-Omi[1], Laura Ortinau[2], and Dongsu Park[2]

[1]*Biologic and Materials Sciences, University of Michigan School of Dentistry, Ann Arbor, United States*

[2]*Molecular Human Genetics, Baylor College of Medicine, Houston, United States*

The coupling of bone resorption to bone formation is critical to maintain bones as a functional organ. Despite recent progress in bone biology, it is still elusive how osteoclasts (OCLs) and osteoblasts (OBs) communicate to maintain bone homeostasis, especially the mode(s) and molecular mechanisms of how OCLs regulate OB function. We have demonstrated that OCLs inhibit OB differentiation via direct cell-to-cell contact, while secreted factors from mature OCLs promote OB mineralization in vitro, suggesting that OCLs regulate OB function via two distinct modes. We have also demonstrated that OCLs physically interact with OBs thorough tunneling nanotubes (TNTs), which transport cargo toward OBs, leading to cell death of OBs in vitro. Here, we further investigated how and when OCLs interact with OBs by intravital imaging and how OCLs induce cell death of OBs via TNTs. To observe real-time OCL-OB interaction, we developed a dual reporter model to label OCL using cathepsin-k-Cre (Ctsk-Cre) and label OBs with osteocalcin-GFP (Ocn-GFP). Ocn-GFP mice were lethally irradiated and received Ctsk-Cre/Tomato donor bone marrow to achieve selective labeling of OCLs. Images from in vivo time-lapse intravital microscopy suggested that OCLs interact with OBs and induce cell death to clean-up bone surfaces at the beginning of bone remodeling cycles. Since these OCLs were migrated away without resorbing bones, it is likely that these is a specific population of OCLs responsible for killing OBs to clean-up bone surfaces. Overall, our in vitro mechanistic studies and in vivo live imaging approaches uncover novel mechanisms of how OCLs regulate OB activities. We believe that outcomes from this study will pioneer a totally new treatment by suppression of TNT formation/function without affecting OCL numbers but to increase bone formation, which is critical to treat human bone-mass related diseases such as osteoporosis.

**THE FINGERPRINT OF BONE RESORPTION: DEFINING THE OSTEOCLAST SPECIFIC SIGNATURE**

Kyung Hyun Park-Min

*SHORE program, Research, Hospital for Special Surgery, New York, United States*

Osteoclasts are large, multinucleated myeloid-derived cells that mediate both physiological and pathological bone resorption. Although osteoclasts are traditionally viewed as terminally differentiated cells with a short lifespan of approximately two weeks, recent evidence indicates that osteoclasts are long-lived and can be continuously replenished through fusion with osteoclast precursor cells (OCPs) and recycling of osteomorphs. Building on this concept, we sought to identify osteoclast-specific cellular and molecular signatures that distinguish these cells from other lineages. We characterized a unique population of circulating osteoclast precursor cells (cOCPs) and identified osteoclast-specific super-enhancer–derived RNAs, termed Oslincs, in human osteoclasts. Dysregulated osteoclast differentiation contributes to excessive bone resorption and underlies diseases such as osteoporosis, rheumatoid arthritis, and lytic bone metastasis. Both Oslincs and cOCPs correlate strongly with osteoclast activity and are perturbed in pathological bone diseases. Collectively, our findings suggest that alterations in these osteoclast-specific signatures may play critical, distinct roles in the pathogenesis of bone diseases and in the regulation of osteoclastogenesis under physiological and pathological conditions.

**LYSOSOMAL TRANSPORTERS IN BONE HEALTH & DISEASE: THE INS AND OUTS OF OSTEOCLASTS**

Nathan Pavlos
*Bone Cell Biology, University of Adelaide, Adelaide, Australia*

Osteoclasts are giant bone-digesting cells that harbour specialised lysosome-related organelles termed secretory lysosomes. Using a proteomic approach, we recently resolved the molecular landscape of osteoclast secretory lysosomes, unmasking several membrane transport proteins (e.g. Slc37a2) as candidate regulators of osteoclast function and the maintenance of skeletal bone mass. Here we report that the lysosomal cysteine importer, major facilitator superfamily domain containing 12 (Mfsd12), is robustly expressed both in human and in mouse osteoclasts where it operates on lysosomes to compartmentalize and store cysteine intracellularly. To investigate the physiological importance of *Mfsd12* in osteoclasts and bone homeostasis, we generated global *Mfsd12* knockout (*Mfsd12*$^{KO}$) mice using a CRISPR-Cas9 genome editing strategy. Although born at sub-Mendelian ratios and reduced in size, *Mfsd12*$^{KO}$ mice were viable to 30 weeks. At 15 weeks, microCT analysis revealed a dramatic low bone mass phenotype consistent with osteoporosis. Strikingly, gross inspection of *Mfsd12*$^{KO}$ viscera uncovered a multisystem disorder, including pale, reduced kidneys harbouring renal calculi, and abnormally enlarged livers. Ultrastructural analyses further revealed a profound lysosomal storage disorder characterised by morphologically distorted lysosomes, some bearing crystals. Altogether, our findings identify Mfsd12 as an essential regulator of lysosome function whose deficiency produces a multisystem lysosomal storage disorder that affects bone homeostasis, and extends to kidneys and the liver, phenotypes that bear resemblance to clinical features observed patients afflicted with the rare lysosomal storage disorder cystinosis.

**SPATIO-FUNCTIONAL HETEROGENEITY OF RESIDENT MACROPHAGES FORMING TISSUE-SPECIFIC CHRONIC INFLAMMATION**

Masaru Ishii

*Graduate School of Medicine, University of Osaka, Osaka, Japan*

Tissue-resident macrophages are present throughout peripheral tissues and exhibit diverse changes in response to their local environment. This diversity, along with alterations in the environment shaping them, can sometimes trigger tissue-specific autoimmune responses. In this presentation I will introduce various autoimmune responses caused by changes in diverse tissue-resident macrophages, especially focusing on the liver and lungs. The liver acts as a gateway where blood from the intestine first enters systemic circulation, creating distinct portal vein and central vein regions, although zone-specific immune regulation has been unclear. Using intravital multiphoton imaging, we found that inflammatory responses are actively suppressed only in the periportal region, leading to identification of a unique population of immunosuppressive macrophages that highly express IL-10. Certain gut microbiota promote the development of these immunosuppressive macrophages, showing that liver immune balance is directly influenced by the microbiota. Disruption of the intestinal barrier worsened inflammation and led to chronic autoimmune liver diseases. Characteristic pathogenic macrophages have been also found in pulmonary fibrosis, demonstrating diverse tissue-resident macrophages serve as triggers for various autoinflammatory responses.

**DYSREGULATED CCL CHEMOKINE SIGNALING AS A SPATIOTEMPORAL DRIVER OF FRACTURE NON-UNION IN HIGH-RISK POPULATIONS: A MULTI-OMICS AND GENOMECHANICAL FRAMEWORK**

Ara Nazarian

*Orthopaedic Surgery, Beth Israel Deaconess Medical Center and Harvard Medical School, Boston, United States*

Background/Purpose: Fracture non-union affects 10–45% of long-bone fractures in aging, diabetic, and obese patients, yet no early biomarkers exist to identify at-risk individuals before irreversible healing failure occurs. CC-motif ligand (CCL) chemokines (CCL2, CCL3, CCL5, CCL7) orchestrate the inflammatory phase of fracture healing by recruiting and spatially positioning immune cells. We hypothesize that CCL dysregulation in comorbid states impairs immune resolution — converting a pro-regenerative inflammatory program into a chronic, non-productive state — and that these signatures are conserved across species and mechanistically linked to biomechanical failure.Methods: Using a rat femoral osteotomy model across healthy, aged, diabetic, and obese cohorts, we are performing bulk and spatial transcriptomics of purified macrophages and T cells at days 1 and 7 post-fracture. Healing outcomes at 4 weeks are assessed via μCT, Raman spectroscopy, and AFM nanoindentation. A tiered blood biomarker strategy combines targeted multiplex CCL assays with discovery proteomics. Cross-species validation integrates multiplex CCL profiling of human fracture plasma from healing and diabetic non-union patients, aligned by healing stage. Early immune signatures are being integrated into a multiscale genomechanical model coupling CCL spatial gradients with finite element modeling of callus mechanics.Results: Preliminary data demonstrate divergent early plasma CCL profiles between union and non- union trajectories, with spatial transcriptomics revealing CCL2 hotspot mispatterning in comorbid callus.Conclusions: This integrated framework links early CCL-driven immune mispatterning to biomechanical failure, establishing a foundation for predictive diagnostics and immune-targeted interventions in high-risk fracture patients.

**HOW INFLAMMASOME-DEPENDENT AND -INDEPENDENT PYROPTOSIS SHAPE BONE PATHOPHYSIOLOGY**

Gabriel Mbalaviele

*Division of Bone and Mineral Diseases, WashU Medicine, gmbalaviele@wustl.edu, United States*

Programmed cell death includes several major forms. Apoptosis is generally immunologically silent because apoptotic cells are rapidly cleared, limiting immune activation. In contrast, pyroptosis, necroptosis, and PANoptosis are lytic and pro-inflammatory. My talk will focus on pyroptosis, a gasdermin-mediated form of programmed cell death. Gasdermins are normally autoinhibited, but cleavage by specific proteases releases the N-terminal domain, which inserts into the plasma membrane and forms pores. When pore formation is extensive, membrane integrity fails, the cell swells, and pyroptosis occurs. Pyroptosis can proceed through two major routes. The inflammasome-dependent pathway is mediated mainly by GSDMD, while inflammasome-independent pyroptosis involves several other gasdermins, including GSDME.

In the first part, I will discuss inflammasome-dependent pyroptosis in bone. This work was motivated by earlier findings that ablation of adiponectin-positive cells increased bone mass, suggesting that inflammatory cell death might contribute to this phenotype. Using a low-dose LPS regimen, we found an unexpected result: LPS induced periosteal bone formation, but not endosteal bone formation. The newly formed bone was later remodeled and integrated into existing bone, improving biomechanical properties. Mechanistically, LPS caused bone marrow inflammation and cell death in both hematopoietic and non-hematopoietic compartments, but not in the periosteum. This suggests that inflammatory conditions suppress endosteal osteogenesis because marrow osteoprogenitors do not survive, while periosteal progenitors remain able to form bone. Lineage-tracing studies supported this interpretation. In control mice, bone marrow-associated osteoprogenitors contributed to endosteal osteoblasts, but after LPS treatment, new bone arose predominantly from periosteal progenitors.
Because LPS activates both the canonical and noncanonical inflammasome pathways, and both converge on GSDMD, we used knockout models to determine which pathway drives this response.

In the second part, I will briefly discuss inflammasome-independent pyroptosis, with a focus on GSDME. We found that GSDME deficiency leads to age-associated bone loss, characterized by increased osteoclastogenesis and reduced bone formation, along with activation of senescence- and necroptosis-related pathways. These findings suggest that GSDME-mediated pyroptosis plays a protective role in skeletal aging and that its loss promotes cellular senescence while potentially shifting cell death toward alternative pro-inflammatory pathways, such as necroptosis, thereby contributing to age-related bone loss.

The take-home message is that pyroptosis can have either beneficial or harmful effects on bone, depending on the gasdermin and biological context.

**MITOCHONDRIAL COMMANDERS: THE NUCLEAR RESPIRATORY FACTOR 1 (NRF1) DRIVES OSTEOCLAST DIFFERENTIATION AND SKELETAL BALANCE**

Carina Scholtysek[1], Agnus Monica Davis[1], Thea Hofmann [1], Ulrike Baschant[2], Brenda Krishnacoumar[3], Jule Taubmann [1], Katrin Palumbo-Zerr[1], Jean-Philippe Auger [4], Gerhard Krönke[4], and Georg Schett[1]

[1] *Department of Internal Medicine 3; Deutsches Zentrum für Immuntherapie (DZI), University of Erlangen-Nuremberg and Universitätsklinikum Erlangen,, Erlangen, Germany*

[2]*Department of Medicine III & Center for Healthy Aging, Medical Faculty, Technische Universität Dresden, Dresden, Germany*

[3]*Medical Faculty, University Hospital, University Duisburg-Essen, Essen, Germany*

[4]*Department of Rheumatology and Clinical Immunology, Charité - Universitätsmedizin Berlin, Berlin, Germany*

Bone-resorbing osteoclasts (OCs) depend on substantial metabolic adaptation, yet the mitochondrial processes that enable their differentiation and sustain bone homeostasis remain insufficiently defined. We identified Nuclear Respiratory Factor 1 (NRF1), a transcription factor essential for mitochondrial biogenesis, as a central regulator of mitochondrial integrity during osteoclastogenesis. Using myeloid-specific NRF1 knockout mice (CX3CR1/NRF1KO) and complementary in vitro models, we demonstrate that NRF1 loss compromises mitochondrial respiration, diminishes metabolic flexibility, and impairs precursor fusion, resulting in markedly reduced OC formation. In vivo, these mitochondrial defects translate into decreased OC numbers, increased trabecular bone mass but reduced cortical thickness, revealing that intact mitochondrial function is indispensable for balanced skeletal remodeling. Together, our findings establish NRF1 as a pivotal transcriptional hub coupling mitochondrial integrity to osteoclast differentiation and bone homeostasis. At the same time, they expose a largely unexplored regulatory landscape: the identity and function of mitochondrial-derived signals that instruct osteoclast fate beyond ATP production. By integrating transcriptional, metabolic, and mitochondrial perspectives, this work provides a mechanistic foundation for targeting NRF1-dependent pathways and opens new avenues to investigate mitochondrial cues as therapeutic entry points in bone related disease.

**INFLAMMATION REVEALS A NOVEL CD115$^{+}$ADGRE4$^{+}$ MYELOID OSTEOCLAST PROGENITOR POPULATION**

Edi Rodak[1,2], Sandra Jastrzebski[1], Ivo Kalajzic[3], Joseph Lorenzo[1], and Sanja Novak[3,4]
[1]*Department of Medicine, University of Connecticut Health Center, Farmington, United States*
[2]*Department of Histology and Embryology, Faculty of Medicine Osijek, Osijek, Croatia*
[3]*Center for Regenerative Medicine and Skeletal Development, University of Connecticut Health Center, Farmington, United States*
[4]*Department of Physiology and Cell Biology, University of Arkansas for Medical Sciences, Little Rock, United States*

Osteoclasts (Ocs) are multinucleated bone resorbing cells, which form by fusion of precursor cells (OCP). We previously identified B220$^{-}$CD3$^{-}$CD11b$^{neg/low}$CD115$^{+}$ (CD115$^{+}$ OCP) cells with high osteoclastogenic capacity during homeostasis. Whether this population contributes to Oc activity in inflammation is unknown.
To identify OCPs during systemic inflammation, we used an established model of TNFa injections over the calvaria in C57Bl/6 mice where, CD115$^{+}$ OCP and B220$^{-}$CD3$^{-}$CD11b$^{neg/low}$CD115$^{-}$ (CD115$^{-}$ OCP) cells were analyzed by scRNA-seq. To assess OCP engraftment and differentiation *in vivo*, we used a novel assay. Cathepsin K Cre mice (CatK-cre$^{+/-}$, donor) were treated with TNF. tdTomato Cre-reporter mice (recipient) were fractured and injected with donor cells. This assay used the unique ability of donor OCPs containing CatK Cre to specifically identify tdTomato-expressing Oc *in vivo* upon fusion with recipient mononuclear cells. Transplant of 100k cells was done on the day of fracture and 10 days post fracture (dpf). Animals were sacrificed at 14 dpf and evaluated histologically for TdTomato signal in the callus.
*In vivo* TNF treatment significantly increased osteoclastogenesis in M-CSF + RANKL treated cultures of BMM and PBMC by 1.5 to 10 fold ($p<0.05$). However, TNF didn't alter osteoclastogenesis in cultured CD115$^{+}$ OCP, suggesting that the increase in Ocs under inflammatory conditions derives from a separate pool of OCPs. Significantly, scRNA-seq analysis demonstrated significant increase (60%) in non-classical (Ly6C$^{-}$Adgre4$^{+}$) monocytes, which were also predominantly CD11b$^{+}$, in the blood and BM of TNF-treated mice. *In vitro*, sorted BM CD115$^{+}$Adgre4$^{+}$ cells formed Ocs at a slower rate than regular BMMs but generated similar bone resorption on bone chips. *In vivo*, transplanted CD115$^{+}$Adgre4$^{+}$ cells fused with endogenous OCP to form TRAP and tdTomato-positive Ocs on the bone surface of callus tissue in erosion lacuna.
These results demonstrate a novel CD115$^{+}$Adgre4$^{+}$ myeloid population of OCPs generating Ocs, which greatly expands in inflammation, circulates and migrates to bone resorption sites, engrafts and fuses with endogenous cells to form Ocs. Targeting these cells could prevent bone loss in inflammatory conditions without affecting homeostatic bone turnover.

**FROM IMAGES TO OMICS: AI-DRIVEN TECHNOLOGIES FOR DECODING THE IMMUNE MICROENVIRONMENT AND GENE REGULATION**

Aristotelis Tsirigos

*Medicine, NYU Grossman School of Medicine, New York, United States*

Understanding biological systems requires integrating information across multiple scales, from tissue morphology to molecular regulation. This talk presents a comprehensive framework of AI-driven technologies that bridge these scales to advance precision medicine. We discuss two complementary approaches: (1) self-supervised learning methods for segmentation-free analysis of multiplex spatial imaging data that characterize tumor microenvironments and identify clinically relevant immune signatures; and (2) deep learning models that predict three-dimensional chromatin architecture and protein-DNA binding from DNA sequence and epigenetic features, enabling high-throughput in silico perturbation screening. Through applications in hematologic malignancies and immune cell dysfunction, we demonstrate how these technologies systematically identify master regulators of disease and therapeutic targets. The integration of AI-driven image analysis with molecular modeling enables patient-specific drug target identification and mechanistic understanding of disease across biological scales, establishing a "virtual cell" platform for precision medicine applications.

**PROGRANULIN PROMOTES NLRP3 INFLAMMASOME-MEDIATED IL-1B PRODUCTION AND SUBSEQUENT INFLAMMATORY BONE LOSS**

Vikrant Piprode[1,2], Liping Wang[1,2], Gurismar Kohli[1], Petronela Nicolae[1,2], Yue Wang[3], Chun Wang[4], Gabriel Mbalaviele[4], Mary Nakamura[5,2], and Robert Nissenson[1,2]

[1]*Division of Endocrinology , San Francisco VA Medical Center, San Francisco, United States*

[2]*Department of Medicine, University of California San Francisco, San Francisco, United States*

[3]*Skaggs School of Pharmacy, University of Colorado, Aurora, United States*

[4]*Division of Bone and Mineral Diseases, Washington University School of Medicine, St. Louis, United States*

[5]*Division of Rheumatology, San Francisco VA Medical Center, San Francisco, United States*

Progranulin (PGRN), a glycosylated protein, is expressed in most tissues, including bones, and individuals with rheumatoid arthritis (RA) have elevated levels in the joints. Recently, we reported that global deletion of Grn (PGRN encoding gene) and microglial/macrophage (MF) specific deletion using Cx3cr1cre protects female mice from aging-induced bone loss. Thus, in the current study, we investigated the role for PGRN in MF-mediated regulation of bone health. We found that both male and female Grn-/- mice exhibited a markedly higher proportion of Mac2Hi (galectin-3) bone marrow MFs (BMMs) with significantly lower Nlrp3 expression. The NLRP3 inflammasome is critical in the generation of bioactive IL-1β and M1 MF polarization. We found that LPS induced- expression of 1) Nlrp3 and Il1b, and 2) M1 MF markers, including iNOS, Il6, Tnfa, and Cxcl10, were down-regulated in Grn-/- mice in both sexes. To understand the clinical relevance, we employed the K/BxN serum transfer–arthritis (STA) model. Notably, Grn-/- mice of both sexes exhibited attenuated inflammation, shown by reduction in paw thickness and arthritic scores. Moreover, Grn-/-mice were significantly protected from STA bone loss in regions surrounding the ankle joints, as assessed by m-CT. Consistently, IL-1β levels in both serum and ankle joint lysates were reduced by approximately 30–50% in Grn-/- mice compared to WT in both the sexes. Collectively, these results suggest that PGRN positively regulates IL-1β production, implying involvement of NLRP3 inflammasomes and demonstrates PGRN’s role in the development of STA. Further flow cytometric analyses revealed that the higher proportions of Mac2Hi BMM subsets in Grn-/- did not change in response to STA and thus, in part, may contribute to resistance to STA. In summary, our findings identify PGRN as a novel regulator of IL-1β production mediated via NLRP3 inflammasomes, which in turn drives arthritic bone loss in mice.

**3D IMAGING OF THE SYNOVIUM DEFINES AN IMMUNOLOGICAL DEFENCE SYSTEM AT THE BLOOD-JOINT BARRIER**

Tetsuo Hasegawa

*Medicine, University of Cambridge, Cambridge, United Kingdom*

Joint pain or inflammation is a common and early feature of a variety of systemic diseases. These include autoimmune diseases, such as systemic lupus erythematosus, as well as infection in organs distant to the musculoskeletal system, including enteric or genitourinary infections, which manifest as reactive arthritis. However, why joints are highly responsive to systemic inflammation and where in the joint the inflammation starts are still unknown. We sought to address these questions by developing a whole mount imaging system of the synovium to profile the vascular, neuronal and immune microarchitecture. This revealed that fenestrated capillaries were specifically located at the lining-sublining interface, in the periphery of the synovium, enabling entry of circulating stimuli into the joint. This area of vulnerability was occupied by three subsets of macrophages that demonstrated distinct responses to systemic immune complex challenge and reciprocally interacted with nociceptor neurons, forming a blood-joint barrier (BJB) to defend joint tissue.

### OSTEOIMMUNOLOGY OF POST-TRAUMATIC OSTEOARTHRITIS IN MICE

Gabriela Loots
*Orthopaedic Surgery, University of California, Davis, Sacramento, United States*

Post-traumatic osteoarthritis (PTOA) is a painful joint disease characterized by the degradation of bone, cartilage, and other connective tissues in the joint. PTOA is initiated by trauma to joint-stabilizing tissues, such as the anterior cruciate ligament, medial meniscus, or by intra-articular fractures. In humans, ~50% of joint injuries progress to PTOA, while the rest spontaneously resolve. To better understand molecular programs contributing to PTOA development or resolution, we examined injury-induced fluctuations in immune cell populations and transcriptional shifts by single-cell RNA sequencing of synovial joints in PTOA-susceptible C57BL/6J (B6) and PTOA-resistant MRL/MpJ (MRL) mice. We identified significant differences in monocyte and macrophage subpopulations between MRL and B6 joints. A potent myeloid-driven anti-inflammatory response was observed in MRL injured joints that significantly contrasted the pro-inflammatory signaling seen in B6 joints. Multiple CD206+ macrophage populations were enriched in MRL injured joints. These CD206+ macrophages also robustly expressed Trem2, a receptor involved in inflammation and myeloid cell activation. Through the use of loss- and gain- of function mouse models we determined that Trem2 expression is in part responsible for the enhanced capacity of MRL joints to be protected from PTOA.

**THE ROLE OF IKBZ AS AN INFLAMMATORY SWITCH IN ARTHRITIS**

Yousef Abu-Amer

*Orthopedic Surgery, Washington University St. Louis, St. Louis, United States*

The transcription factor NF-κB plays a critical role in immune and bone homeostasis. Systemic ablation of key components of NF-κB pathway compromise immune competency and lead to deleterious skeletal anomalies. On the other hand, NF-κB mediates most inflammatory responses. In fact, persistent chronic activity of this pathway is a hallmark of several debilitating musculoskeletal diseases. To decipher targetable components of the pathway that uniquely mediate inflammation while sparing essential functions of NF-κB, we carried transcriptomics studies using synovial cells from murine arthritis models. We identified Nfkbiz (encoding IκBζ) among the top expressed genes. Mechanistically, we documented that synovial cells undergo NF-κB-dependent metabolic shift, leading to glycolytic reprogramming that stabilizes IκBζ, which we identified as the principal pro-inflammatory conduit of NF-κB in synovitis. IκBζ protein levels were elevated in immune cells, synovial fibroblasts, and chondrocytes derived from inflammatory arthritis (IA) and OA; and deletion of Nfkbiz dampened expression of inflammatory mediators and SASPs. Further, global conditional deletion of Nfkbiz attenuated the osteo-inflammatory phenotype of STIA mice. We further found that expression of IκBζ coincided with elevated levels of immune responsive gene-1 (Irg1) in synovial macrophages. Increased expression of Irg1 typically leads to the synthesis and accumulation of intracellular itaconate, a well-established anti-inflammatory metabolite. Administration of cell-permeable form of Itaconate (DI) inhibited inflammatory signals downstream of IκBζ without affecting physiologic NF-κB activity. More importantly, DI efficiently and significantly attenuated progression of STIA in mice. Collectively, pharmacologic inhibition or genetic deletion of IκBζ ameliorate murine OA and IA, establishing IκBζ as a key inflammatory gateway of NF-κB in joint arthritis.

**AXIAL PSORIATIC ARTHRITIS IS PHENOCOPIED IN A HUMANIZED MOUSE MODEL**

Maria Garcia-Hernandez, Madison Brien, Javier Rangel-Moreno , and Christopher Ritchlin

*Allergy, Immunology & Rheumatology, University of Rochester , Rochester , United States*

Background: Psoriatic arthritis (PsA) is a complex and heterogeneous inflammatory disorder affecting the axial skeleton in approximately 30% of patients. The mechanisms underlying psoriatic axial inflammation (AxPsA) are not well understood, and longitudinal studies examining the immune mechanisms involved in disease pathogenesis and progression using human cells in an animal model have not been conducted. Experimental strategy: Two groups of NSG-SGM3 mice were injected with serum and peripheral blood mononuclear cells (PBMC) collected from three biologic naive PsA patients with inflammatory back pain and radiographic or MRI documented sacroiliitis (AxPsA) and PsA without axial disease as controls. At day 30, mice were anesthetized, and their spines were dissected for micro-CT scan analysis. Tissue sections from decalcified spines were stained with anti-CD45, CD14, CD8, Ki67, TNF, and IL-17 antibodies. Results: NSG-SGM3 mice injected with serum and engrafted with PBMC from patients with AxPsA exhibited pronounced spinal deformities. Radiographic imaging revealed marked cervical and thoracic kyphosis, and micro-computed tomography (micro-CT) scans identified both bone resorption and pathological bone fusion in the sacroiliac joint region and spine. In contrast, mice injected with PBMCs and sera from non-AxPsA patients did not exhibit axial structural alterations. Histological analysis in the spine of mice injected with AxSpA PBMC and sera revealed marked accumulation of lymphocytes near the dorsal root ganglion (DRG) and in the surrounding muscle. Inflammatory cell infiltrates were characterized by proliferating CD3 T cells and CD8 T cells that expressed both TNF and IL-17. Conclusion: Injection of PBMC and sera from AxPsA patients recapitulated clinical features in hu PsA mice. Structural damage, including bone loss and new bone formation, was noted in the cervical, thoracic spine, and tail. Proliferating CD3+ T cells and CD8+ T cells expressing RANKL were noted near the DRG and in paravertebral muscles. This model will accelerate the investigation of immune mechanisms in AxPsA.

**OSTEOIMMUNOLOGY OF BONE INFECTIONS**

Gowri Muthukrishnan

*Department of Orthoopaedics, Center for Musculoskeletal Biology, University of Rochester Medical Center, Rochester, United States*

Bone infections remain among the most frustrating and unresolved challenges in orthopaedic surgery, characterized by high recurrence rates, limited treatment guidance, and devastating patient outcomes. Our work aims to define the immune principles that drive chronicity in these infections, with a specific focus on how human T cells behave within the infected bone marrow microenvironment. Using clinically relevant humanized mouse models and patient-derived samples, we uncover a striking, previously underappreciated remodeling of the T cell compartment during Staphylococcus aureus bone infection. Human CD4 T cells accumulate at infected sites as heterogeneous Th1 and Th17 populations that progressively acquire features of functional exhaustion, including elevated expression of immune checkpoint proteins such as TIM-3 and LAG-3, reduced proliferation, and blunted effector cytokine production. Remarkably, these same immune signatures are detectable in patient serum, where high levels of immune checkpoint proteins, particularly TIM-3, strongly predict adverse clinical outcomes. Together, these findings position T cell dysfunction as a central driver of bacterial persistence in bone and identify immune checkpoint pathways as actionable biomarkers of disease severity. This work reframes chronic bacterial osteomyelitis as an immunological failure state and highlights new opportunities to leverage osteoimmunology for improved diagnostics and immune-directed therapies.

**NOVEL ROLES OF TELOCYTES AND MAST CELLS WITHIN THE SYNOVIAL LYMPHATIC SYSTEM, JOINT HOMEOSTASIS, AND INFLAMMATORY-EROSIVE ARTHRITIS**

Edward Schwarz

*Orthopaedics, University of Rochester, Rochester, United States*

Introduction: Lymphatic dysfunction is a hallmark of rheumatoid arthritis (RA) and contributes to chronic joint inflammation and damage[1]. A critical unresolved question is what signals activate mast cells that surround joint-draining lymphatic vessels to regulate lymphatic contractions? Recent scRNAseq analysis of popliteal lymphatic vessel (PLV) cells [2] identified Efhd1 as a selective marker of a previously unrecognized peri-PLV telocyte population. Telocytes are stromal cells with long telopodes that support tissue organization and cell communication. Notably, these telocytes are significantly reduced in RA synovium [3]. Transmission electron microscopy and confocal microscopy further revealed direct contacts between mast cells and telocyte networks, suggesting that these peri-PLV telocyte networks sense osmotic and mechanical stress in the joint and signal to peri-PLV mast cells that release vasoactive molecules to modulate lymphatic function.

Methods: PLVs from tamoxifen-treated Efhd1-CreERT2 x Ai9 mice were cultured ex vivo to establish co-cultures of tdT+ telocytes and tdT- fibroblasts. Osmotic stress responses were assessed using sucrose, with Fluo-4 and MitoSOX-Green dyes measuring Ca++ flux and mitochondrial ROS respectively. We also performed Matrigel invasion assays +/- TGF-β, and bulk RNAseq of FACS purified $tdT^+$ telocytes cultured on low, intermediate, and high stiffness-defined extracellular matrix (ECM) substrates.

Results: Telocytes exhibited significantly greater Ca++ flux in response to sucrose-induced hyperosmotic stress than fibroblasts (63.83% ± 6.86% vs 45.95% ± 5.33%, $p<0.01$), and increased mitochondrial ROS production was only observed in telocytes (10min vs baseline, $p<0.0001$). Fibroblasts displayed greater invasive capacity in Matrigel vs. telocytes (209.80 ± 17.38 µm vs 183.06 ± 28.26 µm). Bulk RNAseq showed tdT+ telocytes differentiated into myofibroblasts on stiff ECM. Consistently, scRNAseq trajectory analysis of synovial lymphatic system (SLS) cells predicted telocyte-to-fibroblast differentiation, supporting their potential contribution to pathogenic fibroblast populations.

Discussion: Our findings support a model in which Efhd1+ telocyte networks regulate joint homeostasis by sensing osmotic stress and relaying signals to mast cells, thereby promoting lymphatic clearance. Mechanistically, telocytes exhibit high cytosolic Ca++ flux and increased mitochondrial ROS (Mitoflash), coupled with low invasive capacity, consistent with Efhd1 mediated regulation of mitochondrial Ca++ uptake and inhibition of YAP/TAZ metastasis pathway [4]. In contrast, under conditions of chronic inflammation and stiff ECM, telocytes lose Efhd1 expression, acquire myofibroblast features, and activate the YAP/TAZ pathway enhancing invasiveness. Collectively, our study identifies telocytes as a critical stromal population that senses osmotic and mechanical cues to regulate SLS functions, which highlights their potential as therapeutic targets to restore joint drainage and limit damage from arthritis.

1. Bouta, E.M., et al., 2018. 14(2): p. 94-106.
2. Kenney, H.M., et al., Arthritis Res Ther, 2022. 24(1): p. 64.
3. Rosa, I., et al., J Cell Mol Med, 2021. 25(4): p. 2274-2278.
4. Meng, K., et al., Cancer Sci, 2023. 114(5): p. 2029-2040.

**AGE-RELATED MECHANISMS OF ALPHAVIRUS-MEDIATED ARTHRITIS – LESSONS FOR TISSUE REMODELING IN OLDER AGE**

Janko Nikolich

*Department of Basic Medical Sciences and the Arizona Center on Aging, University of Arizona College of Medicine-Phoenix, Phoenix, United States*

Chikungunya virus (CHIKV) is a re-emerging global pathogen with pandemic potential, which causes fever, rash and debilitating arthritis with arthralgia. Older adults over 65 years are particularly susceptible to severe and chronic CHIKV disease (CHIKVD), accounting for >90% of all CHIKV-related deaths. There are currently no approved vaccines or antiviral treatments available to limit chronic CHIKVD. We found that in old mice excessive, dysregulated TGF-beta production during acute infection leads to a reduced immune response and subsequent chronic disease. Humans suffering from CHIKV infection also exhibited high TGF-beta levels and a pronounced age-related defect in neutralizing anti-CHIKV antibody production. In vivo reduction of TGF-beta levels minimized acute joint swelling, restored neutralizing antibody production and diminished chronic joint pathology in old mice. This study identifies increased and dysregulated TGF-beta secretion as one key mechanism contributing to the age-related loss of protective anti-CHIKV-immunity leading to chronic CHIKVD. We discuss these results in light of our other findings showing that infections with other RNA viruses, as well as normal aging of secondary lymphoid organs, carry pronounced signatures of hyperactivity of the TGF-beta pathway, conducive to fibrotic lesions.

**THE CONTRIBUTION OF DECTIN-1 TO PERIODONTAL BONE LOSS**

Gulnaz Guliyeva

*Department of Oral and Cranio-Maxillofacial Surgery., Friedrich-Alexander University Erlangen-Nürnberg (FAU)., Erlangen, Germany*

The contribution of Dectin-1 to Periodontal Inflammation Gulnaz Guliyeva1,2, Roman Gerlach2, Leah Trumet3, Margarete Schimpf2, Georg Schett1, Aline Bozec1, Manuel Weber2, Bettina Grötsch1 1 Department of Internal Medicine 3 – Rheumatology and Immunology, Friedrich-Alexander University Erlangen-Nürnberg (FAU), Erlangen, Germany 2 Department of Oral and Cranio-Maxillofacial Surgery, Friedrich-Alexander University Erlangen-Nürnberg (FAU), Erlangen, Germany 3 Department of Operative Dentistry and Periodontology, Uniklinikum Erlangen, Germany Periodontitis is a chronic inflammatory disease driven by dysbiotic biofilms and characterized by progressive destruction of the tooth-supporting tissues. It affects over half a billion people worldwide. and is the leading cause of tooth loss in older adults. Bone loss is primarily mediated by osteoclasts, whose differentiation and activation are driven by the interaction between the receptor RANK (receptor activator of nuclear factor-kB) and its ligand, RANKL. Dectin-1 (Clec7a) is a C-type lectin receptor expressed by myeloid cells and functions as a pattern recognition receptor for bacterial and fungal β-glucans. Given that osteoclasts originate from the monocyte–macrophage lineage and depend on ITAM/SYK signaling pathways downstream of C-type lectin receptors, we hypothesize that Dectin-1 serves as a critical link between microbial infection and pathological bone loss in periodontitis. Supporting this concept, our preliminary data show that Dectin-1 (clec7a) expression is upregulated in human (1.337 vs. 4.465 rel. expression to actin p=0.023) and murine jawbone derived (0.0003 vs. 0.002 rel. expression to actin p=0.02) osteoclast precursor cells in response to ß-glucan stimulation. In addition, Dectin-1 activation induced the number of polynucleated wt jawbone osteoclasts in vitro (30.63 vs.52.14 p=0.0047). By using the mouse model of ligature periodontitis, µCT data show that Clec7a-/- mice are partially protected from bacterial induced bone loss with a significant increased bone volume and a decreased dental pocket (distance between tooth root and bone (121.1µm vs. 83.4µm p

**LOOKING THROUGH A DEVELOPMENTAL LENS TO BETTER UNDERSTAND THE OSTEOSARCOMA TUMOR MICROENVIRONMENT**

Elizabeth Young, Christine Johnson, Alex Lee, and Alejandro Sweet-Cordero
*Pediatrics, University of California San Francisco, San Francisco, United States*

Patients with metastatic osteosarcoma (OS) have dismal outcomes that have thus far not been improved by current immunotherapies, highlighting the need for new, immunobiology-driven treatments. I hypothesize that the immunosuppressive, myeloid-rich tumor microenvironment (TME) of OS represents a targetable vulnerability arising from "developmentally hijacked" interactions between OS progenitors and myeloid precursors during tumor evolution in the trabecular region of long bones. A pathogenic role for OS-derived cytokines including IL-6, CCL2, CCL5, M-CSF, and CXCL10 is supported their robust secretion from OS patient-derived xenograft (PDX) cell lines, which shape bone marrow monocyte-derived macrophages toward an "M2-like" suppressive phenotype ($MHCII^{-}CD206^{+}$). In co culture and in vivo OS models, I have shown that STING agonism-mediated Type I interferon signaling reverts suppressive myeloid programs, leading to tumor regression and lasting immunologic memory independent of tumor STING. To investigate involvement of the myeloid compartment in this therapeutic response, I performed scRNA-seq on K7M2.1 murine orthotopic OS tumors. Among immune cells, monocytes and macrophages were most enriched in treated samples. Notably, our previously derived STING activation signature was most enriched in monocytes within treated tumors, reinforcing the critical role of this tumor-infiltrating myeloid population in mediating STING activation responses. Future work will identify the key cytokines that initiate a developmental program resulting in an immunosuppressive, myeloid-rich TME and determine the mechanism by which STING agonism overcomes these myeloid programs that are established during OS development. Understanding the developmental mechanisms that establish and maintain the OS TME is essential for translating novel immunotherapies to improve outcomes for this aggressive pediatric cancer.

**MECHANICAL REGULATION OF STRUCTURAL AND METABOLIC REMODELING IN TENDON DURING AGING AND ENVIRONMENTAL ADAPTATION**

Takahiro Morita[1,2], Tomoki Chiba[1], Takahide Matsushima[1], Ryo Nakamichi[3], Yuta Fujii[1,2,4], Kenji Takahashi[2], and Hiroshi Asahara[1,4]

[1]*Systems BioMedicine, Institute of Science tokyo, Tokyo, Japan*

[2]*Orthopaedics, Kyoto Prefectural University of Medicine, Kyoto, Japan*

[3]*Orthopaedic Surgery/Faculty of Medical Development Field, Okayama University , Okayama, Japan*

[4]*Molecular and Cellular Biology, Scripps Research, La Jolla, United States*

Mechanical loading regulates homeostasis in musculoskeletal tissues. How mechanotransduction intensity influences age-associated tissue remodeling and its relevance to immune–skeletal interactions remains unclear. We performed bulk RNA sequencing of Achilles tendons from wild-type mice at 18 and 100 weeks of age. Aging was associated with downregulation of extracellular matrix (ECM) organization and collagen metabolic processes, accompanied by suppression of glycolytic pathways and increased oxidative phosphorylation and mitochondrial ATP synthesis. These findings indicate a shift from a glycolysis-associated matrix-producing state toward a more oxidative metabolic program during tendon aging.In tendon-specific Piezo1 gain-of-function mice, bulk RNA sequencing showed relative preservation of tendon lineage gene expression compared with wild-type controls during aging, and locomotor performance was maintained. Environmental mechanical conditions also influenced transcriptional programs. In young wild-type mice housed on substrates of differing rigidity for four weeks, hard substrates increased collagen-related gene expression in Achilles tendon. In medial collateral ligament tissue, hard environments were associated with increased mitochondrial metabolic pathways and changes in extracellular matrix and leukocyte migration gene signatures. These results suggest that genetic enhancement of mechanotransduction and environmental mechanical loading reshape structural and metabolic programs in musculoskeletal tissues during aging and mechanical adaptation.

**ESTABLISHING AN IN VITRO APPROACH TO GENERATE OSTEOMORPHS**

Christiane Schirm, Martina Rauner, and Lorenz Hofbauer

*Medizinische Klinik und Poliklinik III, University Hospital Dresden, Technische Universität Dresden, Dresden, Germany*

Osteoporosis is driven by increased osteoclast activity resulting from e.g. inflammation, estrogen deficiency, or glucocorticoid treatment. While the anti-RANKL antibody denosumab effectively increases bone density, its discontinuation triggers a "rebound effect" with rapid osteoclast formation and high fracture risk. The recently described osteomorphs—cells derived from osteoclast fission—are suspected mediators of this process. This study aims to establish a cell culture model to characterize osteomorphogenesis and its regulation. Osteoclasts were generated from wild-type and reporter mice (mT/mG, Vav:cre-mT/mG). Differentiation, fission, and re-fusion dynamics were monitored via PhC microscopy. Cellular identity of osteoclasts was validated using TRAP staining (positive cells with at least three nuclei), qRT-PCR (e.g. Ctsk, Nfatc1, Rank), and flow cytometry (FACS) to distinguish osteomorphs from other myeloid lineages. Re-fusion capacity was tested using fluorophore-labeled cells and viability assays. RANKL stimulation (50 ng/mL) induced osteoclast formation over 5–6 days. Subsequent RANKL withdrawal for 2 days triggered osteoclast recycling; approximately 65% of supernatant-derived cells remained viable and were declared as “osteomorph-like”. FACS characterized these cells as $CD45^{+}$ $CD115^{-}$ $RANK^{+}$ $CD11b^{+}$ $Ly6c^{+}$. Gene expression analysis showed a downregulation of osteoclast markers and upregulation of proinflammatory cytokines. Although the transcription factor Nfatc1 decreased, it remained detectable, and Rank expression persisted. Upon RANKL re-stimulation, these cells re-fused into TRAP-positive osteoclasts. Notably, these re-fused cells exhibited higher marker gene expression and a larger total surface area compared to initial osteoclasts. Fluorescence microscopy confirmed re-fusion by identifying double-positive cells from differentially labeled populations. Taken together, we established an in vitro protocol to simulate osteoclast-osteomorph-recycling events.

**CD14ST AND OSTEOIMMUNOLOGICAL BIOMARKERS : EFFECTS OF COVID19 ON BONE REMODELING**

Emanuela Galliera[1,2], Luca Massaccesi[1,2], Laura Mangiavini[1,2], Giuseppe Peretti[1,2], and Massimiliano Corsi Romanelli[1,3]

[1]*Department of Biomedical Sciences for Health, University of Milan , Milan , Italy*

[2]*IRCCS Galeazzi Orthopedic Institute, Milan, Italy, IRCCS Galeazzi Orthopedic Institute, Milan , Italy*

[3]*Department of Experimental and Clinical Pathology, IRCCS Istituto Auxologico Italiano, Italy, IRCCS Istituto Auxologico Italiano, Italy, Milan, Italy*

Background/Objectives: . The COVID-19 pandemic has raised interest in osteoimmunology because of the impact of SARS-CoV-2 on both the immune system and the bone microenvironment. Soluble CD14ST could influence the production of osteoimmunological regulators of osteoclast differentiation. This study aims to evaluate the role of sCD14ST in the COVID-19-induced effects on bone remodeling, particularly by examining the correlation with new-generation osteoimmunological biomarkers, and to gain a comprehensive understanding of the disease's impact on the immune and skeletal systems. Methods: The Serum level of sCD14ST was measured in COVID-19-positive and negative patients undergoing orthopedic surgery and correlated with the inflammatory and osteoimmunological biomarkers RANKL/OPG, FGF23, IL-6, CRP, PCT, sRAGE, and SuPAR. Results: In our patients, sCD14ST showed a strong increase in COVID-19-positive patients and a significant decrease along with the infection resolution, confirming its diagnostic and prognostic value. sCD14ST resulted more clinically relevant than the two canonically inflammatory makers used in the clinical protocols, CRP and PCT, and it displayed a good positive correlation with FGF23, RANKL/OPG, IL-6, SuPAR and a negative correlation with sRAGE. Conclusions: Monitoring sCD14ST, along with SuPAR, may offer valuable insight into immune system dysregulation and bone-related complications in conditions characterized by inflammation. These soluble receptors represent important links between immune activation and bone metabolism, especially in the context of diseases like COVID-19, where the inflammatory response may impact bone fragility.

### A BIOPRINTED JOINT-ON-A-CHIP PLATFORM FOR MODELLING OSTEOIMMUNE INTERACTIONS IN INFLAMMATORY ARTHRITIS

Danka Grcevic[1,2], Alan Sucur[1,2], Darja Flegar[1,2], Sara Priselac[2,3], Dominik Lukicic[3], Tomislav Balen[2,3], Ivo Kresic[4], Amir Secerkadic[5], Marko Peric[5], and Natasa Kovacic[2,3]

[1]*Department of Physiology and Immunology, University of Zagreb School of Medicine, Zagreb, Croatia*

[2]*Croatian Institute for Brain Research, University of Zagreb School of Medicine, Zagreb, Croatia*

[3]*Department of Anatomy, University of Zagreb School of Medicine, Zagreb, Croatia*

[4]*Department of Physiology, University of Mostar School of Medicine, Mostar, Bosnia and Herzegovina*

[5]*3D Printing, Izit d.o.o., Sveta Nedelja, Croatia*

Pathogenesis of inflammatory joint diseases involves complex interactions within bone, cartilage, and synovium. Traditional 2D cultures and animal models fail to recapitulate the human 3D microenvironment, limiting therapeutic predictive value. We aim to develop a modular joint-on-a-chip platform combining 3D-printed biocompatible supports with bioprinted tissues to study inflammatory cell infiltration and drug responses in arthritis. Biocompatible resins (MED610, MED620) were 3D-printed and optimised via post-processing. Cell-laden constructs ($1\times10^6$ cells/mL) were bioprinted using BioX6 with adECM-, GelMA-, and collagen-based bioinks. Human mesenchymal (hBMSC, HS27a), synovial (SW982), endothelial (HUVEC) lines, and osteoarthritis patient-derived primary cells were used. Viability (annexinV/7-AAD) and phenotype (CD73, PDPN, CD146, CD140, CD105) were assessed by flow cytometry. Osteogenic differentiation was evaluated by alizarin red and RUNX2, ALP, BGLAP; chondrogenic by alcian blue and SOX9. Monocyte migration through Transwell-based adECM toward chemokines (CCL2, CCL5, CXCL10) was assessed. MED610/MED620 supported OchP survival, proliferation and osteogenic differentiation. adECM outperformed commercial inks with higher OchP viability, altered RANKL/OPG ratio, and enhanced RUNX2/SOX9 expression. Histology showed lacuna-like structures resembling native cartilage. Primary cells exhibited greater heterogeneity and reduced differentiation versus immortalized lines. HUVEC-mesenchymal co-cultures formed capillary-like cords. adECM

### ALTERING HOST MICROENVIRONMENT IS CRITICAL FOR SUCCESS OF CELL THERAPY FOR OSTEOGENESIS IMPERFECTA

Sanja Novak[1,2], Ying Tang[2], Milos Opacic[2], Alexandra Marinescu[2], Namrata Kanwal[2], and Ivo Kalajzic[2]

[1]*Department of Physiology and , UAMS, Little Rock, United States*

[2]*Center for Regenerative Medicine and Skeletal Development, UConn Health, Farmington, United States*

Current treatments for osteogenesis imperfecta (OI) do not address the underlying defect in collagen production. Transplantation of mesenchymal stem/stromal cells (MSCs) directly into bone presents a promising strategy for a cellular replacement therapy. Unlike systemic transplantation, locally delivered MSCs can engraft efficiently, differentiate into matrix-producing osteoblasts, and generate normal collagen. We aimed to determine how alterations in the bone marrow microenvironment influence MSC engraftment and their differentiation. Different precondition treatments to achieve successful transplantation were explored: (I) lethal (900 cGy) and sublethal (500 cGy) irradiation (IRR), (II) myeloablation using Busulfan and Cyclophosphamide (BuCy), (III) decreasing bone turnover with zoledronic acid, and (IV) osteoblast depletion using specific cell ablation model. When using IRR, a day before BMSCs transplantation (106), hematopoietic cells were reconstituted. Cell engraftment was evaluated 30 days post-transplantation. Lethally irradiated OIM mice exhibited highest donor cell engraftment on the endosteal surface compared to sublethal and non-IRR mice. Myeloablation led to similar changes in T, B and myeloid (CD11b+) cell composition as IRR (3 days post transplantation) while decreased proportion of NK (69%) and neutrophils (27%) compared to IRR group, resulting in much lower engraftment rate (60% less osteoblasts). Donor cells are almost absent in control OIM animals without IRR and osteoblast ablated OIM/Col2.3ΔTK/+ mice. However, IRR led to comparable cell engraftment with or without osteoblast ablation (OIM/Col2.3ΔTK/+: 32%, OIM: 34% osteoblasts on endosteal bone). Zoledronic acid reduced endosteal surface area occupied by donor osteoblasts, suggesting importance of high bone turnover for donor cell engraftment. Interestingly, IRR and BuCy myeloablation increased bone marrow adipogenesis, by directing recipient mesenchymal progenitors into adipogenic lineage (98% adipocytes are host derived). Lethal irradiation is the effective pretreatment for MSCs engraftment in bone and their differentiation into osteoblasts producing healthy matrix. Directing hosts MSCs to adipocytes, along with donor MSCs differentiation into osteoblasts is a likely mechanism. Our results inform future strategies on how to improve the efficacy of MSCs-based therapies for OI.

**ANNEXINA1-DECTIN 1 AXIS IS A KEY REGULATOR OF OSTEOCLASTOGENESIS UNDERLYING IRRADIATION INDUCED BONE LOSS**

Gulnaz Guliyeva[1], Anja Derer[2], Benjamin Frey[2], Pooja Gupta[3], Manuel Weber[4], Leah Trumet[5], Georg Schett[1], Aline Bozec[1], and Bettina Groetsch[1]

[1]*Department of Medicine 3 – Rheumatology and Immunology, Uniklinikum Erlangen, Erlangen, Germany*

[2]*4Translational Radiobiology, Department of Radiation Oncology, Uniklinikum Erlangen, Erlangen, Germany*

[3]*5Department of Stem Cell Biology, Uniklinikum Erlangen, Erlangen, Germany*

[4]*6Department of Oral and Cranio-Maxillofacial Surgery, Uniklinikum Erlangen, Erlangen, Germany*

[5]*7Department of Operative Dentistry and Periodontology, Uniklinikum Erlangen, Erlangen, Germany*

Bone homeostasis depends on the efficient clearance of apoptotic cells by phagocytes. While cell death is a physiological component of hematopoiesis and bone remodeling, anticancer radiotherapy induces excessive apoptosis and inflammation, ultimately contributing to bone fragility. Recognition of apoptotic cells is mediated through phosphatidylserine, either directly or via bridging molecules such as AnnexinA1 (AnxA1). Dectin-1 (CLEC7A), a myeloid C-type lectin receptor best known for β-glucan sensing, also binds endogenous ligands including AnxA1. Since AnxA1 is highly expressed on apoptotic immune cells and its levels increase following radiation-induced cell damage, it is likely that AnxA1 interacts with Dectin-1 to regulate osteoclast differentiation in radiation-induced bone loss. We identify an AnxA1–Dectin-1 signaling axis as a key driver of osteoclast differentiation. Dectin-1 deficient mice exhibited significantly reduced osteoclast numbers (N.OC/B.Pm: 3.92 vs. 2.87 1/mm; p=0.0018) and increased trabecular bone volume (BV/TV: 0.12 vs. 0.18%; p=0.02). In vitro, β-glucan–induced Dectin-1 activation enhanced osteoclastogenesis in a Dectin-1–dependent manner (30.6 vs. 52.1 OC > 5 nuclei; p=0.002). Within the bone marrow niche, AnxA1 was abundantly expressed on $B220^+$ B cells, and γ-irradiation markedly increased AnxA1 surface translocation both in vitro (16.6 vs. 36.6% AnxA1; p < 0.0001) and in vivo (7.2 vs. 20.2% AnxA1; p< 0.0001). Following irradiation, $B220^+$ B cells displayed increased Dectin-1 binding and promoted multinucleated osteoclast formation, which was abrogated by Dectin-1 deficiency. RNA-seq analysis of wild-type versus clec7a-/- pre-osteoclasts co-cultured with irradiated $B220^+$ cells revealed selective activation of MAPK, PI3K, calcium, and Rap1/Raf signaling pathways exclusively in wild-type cells. Functionally, clec7a-/- mice were protected from radiation-induced bone loss: µCT showed reduced BV/TV only in irradiated wild-type mice (BV/TV: 0.16 vs. 0.10%; p=0.048), accompanied by increased osteoclast surface and osteoclast number (N.OC/B.Pm: 5.3 vs. 6.6 1/mm; p=0.003). In contrast, clec7a-/- mice exhibited no changes post-irradiation (BV/TV: 0.2 vs. 0.16%; p = 0.09; N.OC/B.Pm: 4.5 vs. 4.7 1/mm; p=0.8). Our findings uncover AnxA1–Dectin-1 signaling as a crucial immune–skeletal communication pathway through which radiation-exposed immune cells accelerate bone resorption. Targeting this axis may provide a therapeutic strategy to mitigate radiation-induced bone deterioration.

### ANTIRESORPTIVE THERAPY REWIRES OSTEOIMMUNE SIGNALING IN MRONJ: THE ROLE OF ANTIMICROBIAL PEPTIDES IN BONE TURNOVER IMBALANCE

Linus Winter[1], Annika Haeuser[2], Stella Luckey[2], Agnus Davis[2], Sofia Hutter[2], Susnigdha Sen[2], Matthias Kuebler[1], Cornelia Stoll[2], Alexandra Correa Zamora[2], Manuel Weber[1], Georg Schett[2], Marco Kesting[1], and Carina Scholtysek[2]

[1]*Department for Oral and Cranio-Maxillofacial Surgery, University Hospital Erlangen, FAU Erlangen-Nürnberg, Erlangen, Germany*

[2]*Department of Internal Medicine 3 Rheumatology and Immunology, University Hospital Erlangen, FAU Erlangen-Nürnberg, Erlangen, Germany*

Antiresorptive (AR) therapies, including bisphosphonates and denosumab, effectively reduce skeletal-related events but may cause medication-related osteonecrosis of the jaw (MRONJ), a rare yet severe complication. Increasing evidence suggests that MRONJ is not solely a bone-centered disorder but arises from disrupted osteoimmune homeostasis within the jaw, where pharmacologically altered bone turnover intersects with microbial dysbiosis and impaired mucosal immunity. Mucosal integrity critically depends on commensal-induced antimicrobial peptides (AMPs), which not only mediate microbial defense but also regulate inflammation, barrier function, angiogenesis, metabolism, and osteoclast–osteoblast communication. Recent studies highlight the cathelicidin LL-37 (murine ortholog CRAMP) as a key regulator at the interface of mucosal immunity and bone remodeling. Our data demonstrate that LL-37 is elevated in MRONJ tissue samples and at oral infection sites. In contrast, previous studies have reported dysregulated LL-37 levels in chronic periodontitis, which may be associated with periodontal breakdown and alveolar bone loss. Further, our data identify CRAMP as a central osteoimmune mediator: CRAMP promotes osteoblast mineralization, inhibits osteoclast differentiation and resorptive activity, reduces mitochondrial metabolic function in osteoclasts, and disrupts the NRP1/SEMA3A signaling axis, a critical pathway in OC–OB crosstalk and skeletal homeostasis. Importantly, we observed that the anti-inflammatory effects of LL-37 may reverse in the presence of zoledronate, leading to paradoxical proinflammatory responses. This finding suggests that local BP exposure can fundamentally reprogram AMP function, potentially shifting immune responses from protective to pathogenic. We therefore hypothesize that AR therapy, in conjunction with oral dysbiosis, reprograms the expression and functional activity of AMPs such as CRAMP/LL-37. This shift in AMP-mediated signaling may disrupt osteoclast–osteoblast coupling, impair physiological bone turnover, weaken mucosal defense, and foster a proinflammatory microenvironment that predisposes to jaw necrosis. We further propose that distinct microbiome–AMP interaction profiles may underlie individual susceptibility to MRONJ.

**BRIDGING IMMUNITY AND BONE: DECTIN-1–RANK SIGNALING REGULATES HUMAN OSTEOCLAST DIFFERENTIATION**

Marina Kleim[1], Aline Bozec[1], Manuel Weber[2], and Bettina Grötsch[1]

[1]*Department of Internal Medicine 3 – Rheumatology and Immunology, Friedrich-Alexander University Erlangen-Nürnberg (FAU), Erlangen, Germany*

[2]*Department of Oral and Cranio-Maxillofacial Surgery, , Friedrich-Alexander University Erlangen-Nürnberg (FAU), Erlangen, Germany*

Bone remodeling is a continuous process that depends on the balance between bone for- mation and bone resorption. RANK signaling, driving osteoclastogenesis alongside M-CSF, depends on ITAM signaling of a co-stimulatory receptor. The C-type lectin receptor Dectin-1, known for its role in antifungal immunity, contains such an ITAM-like motif which can synergize with RANK signaling, highlighting its potential involvement in osteoclastogenesis, bridging im- mune sensing with bone metabolism.

The aim was to establish a Dectin-1 knockout model in human monocytes, to investigate the influence of β-glucan-induced Dectin-1 signaling on the differentiation and function of human osteoclasts *in vitro* and in periodontitis.

The CRISPR/Cas9-mediated knockout of Dectin-1 markedly reduced the formation on multi- nucleated osteoclast in response to β-glucan stimulation (1.42 vs 0.62 cell count p=0.002), while resorptive activity was mostly maintained. Additionally, Seahorse extracellular flux anal- ysis revealed that Dectin-1-KO osteoclasts exhibited a significant decline in mitochondrial (796.22 vs 2505.03 OCR p=0.04) and a reduction in glycolysis (60.56 vs 121.1 ECAR p=0.034) and glycolytic capacity (74.77 vs 148.64 ECAR p=0.004), suggesting that Dectin-1 contributes to the metabolic reprogramming required for efficient osteoclast differentiation. Further, in the blood of periodontitis patients higher levels of Dectin-1 (34.95 vs. 45.67 percent p=0.048; 0.006 vs. 0.011 relative expression p=0.0035) were measured. Osteoclasts derived from patients demonstrated also enhanced differentiation (2.13 vs 1.16 cell count p=0.024) and resorptive activity (1.28 vs 0.88 resorbed area p=0.002) compared with healthy controls with a notably stronger effect observed in female patients (female: 1.00 vs 2.69 cell count p=<0,0001; 1.00 vs 3.11 resorbed area p=0.042; males: 1.00 vs 1.13 cell count; 1.00 vs 0.96 resorbed area) indicating possible sex-specific differences in osteoclast activity in inflammatory bone loss.

These findings identify Dectin-1 as a regulator of osteoclast differentiation and metabolism, by integrating receptor activation by β-glucans with RANK signaling.

**CHARACTERIZATION OF A PRO-RESOLVING EOSINOPHIL SUBSET IN RHEUMATOID ARTHRITIS AND ITS REGULATION BY ANTI-RHEUMATIC TREATMENT**

Nagham Al Arawe[1,2], Md Adnan Karim[1,2], Andrea Zoli[3], Katerina Kachler [1,2], David Barinberg [4], Arif Ekici [5], Aleix Rius Rigau[1,2], Laura Bucci [1,2], Carlo Tur[1,2], Maria Gabriella Raimondo[1,2], Fabian Hartmann[1,2], Georg Schett [1,2], and Aline Bozec [1,2]

[1]*Department of Internal Medicine 3 – Rheumatology and Immunology, Universitätsklinikum Erlangen, Erlangen, Germany*

[2]*Deutsches Zentrum Immuntherapie (DZI), Friedrich-Alexander- Universität Erlangen-Nürnberg , Erlangen, Germany*

[3]*Department of Rheumatology, Fondazione Policlinico Universitario A Gemelli, IRCSS, Catholic University of Sacred Heart, Rome, Italy*

[4]*Institute for Clinical Microbiology, Immunology and Hygiene, Universitätsklinikum Erlangen, Erlangen, Germany*

[5]*Institute of Human Genetics, Universitätsklinikum Erlangen, Erlangen, Germany*

Rheumatoid arthritis (RA) is a chronic inflammatory autoimmune disease characterised by persistent synovitis, impaired inflammation resolution and structural damage. Although the pro-inflammatory cytokines and mediators that control RA are well understood, the factors that control the resolution of inflammation are less well known. Eosinophils, which were once considered to be terminal effector cells in helminth infections and allergic diseases, are now recognised as being key pro-resolving regulators. In murine arthritis, resolving eosinophils (rEos) polarise macrophages towards anti-inflammatory phenotypes and protect bone by inhibiting osteoclast formation via eosinophil peroxidase-dependent crosstalk. The role of eosinophils in human RA remains to be defined. Using flow cytometry, SCENITH, CyTOF, bulk RNA sequencing and single-cell RNA sequencing, we are investigating how different subset of eosinophils accumulates in human RA during remission. Furthermore, anti-rheumatic therapies independently modulate eosinophil states and thereby influence disease resolution. Our flow cytometry data revealed significant upregulation of the rEos markers CD62L and CD274 in patients in remission, compared with healthy controls (HC). UMAP analysis of a multi-marker panel revealed disease state-dependent restructuring of the eosinophil state in RA. Eosinophils from RA patients in remission were shown to be more glucose-dependent and less metabolically flexible than eosinophils from healthy individuals. Additionally, our scRNA-seq data revealed that eosinophils from RA patients in remission upregulate genes related to the regulation of inflammation compared to eosinophils from HCs. Furthermore, our bulk RNA-seq data revealed a set of genes that are potentially responsible for inducing remission and are upregulated in RA patients in remission compared to those with active RA. Interestingly, when remission samples are grouped by current treatment, the mean fluorescence intensity (MFI) of CD62L is significantly higher in patients receiving JAK inhibitors than in RA remission patients receiving bDMARDs or csDMARDs. Altogether, we identify a subset of eosinophils associated with the resolution of RA that express high levels of CD62L and CD274, and have distinct molecular and functional profiles in the blood and synovium of RA patients in remission.

**CSF1R⁻ MACROPHAGES GOVERN THE SECOND WAVE OF NEUTROPHILS FOR BONE REPAIR**

Zhifeng He[1], Linan Shi[2], Yuko Nakamichi[1], Kohei Murakami[3], Toshihide Mizoguchi[4], Yuki Matsushita[5], Hiroshi Takayanagi[6], Nobuyuki Udagawa[1], and Yasuhiro Kobayashi[1]

[1]*Hard Tissue Research, Matsumoto Dental University, Matsumoto, Japan*

[2]*Stomatology, Westlake University, Hangzhou, China*

[3]*Veterinary Immunology, Okayama University of Science, Okayama, Japan*

[4]*Oral Science, Tokyo Dental College, Tokyo, Japan*

[5]*Skeletal Development and Regenerative Biology, Nagasaki University Graduate School of Biomedical Sciences, Nagasaki, Japan*

[6]*Immunology, The University of Tokyo, Tokyo, Japan*

Macrophages (Mφs) play a pivotal role in tissue regeneration, including bone healing; however, the mechanisms and cell types through which they establish regenerative niches are not yet fully understood. Here, we identified a distinct subset of osteodirective Mφs (OdMacs), characterized by the expression profile F4/80$^{+}$ Csf1r$^{-}$ Cx3cr1hi Ccr2hi, which contribute to the formation of bone regenerative niches following injury. Histological analysis and FACS analysis, combined with transcriptomic profiling (RNA-seq and scRNA-seq), revealed that OdMacs facilitate the recruitment of activin A-expressing preneutrophils (preNeu) to sites of bone injury. This recruitment enhances the proliferation and osteogenic differentiation of LepR$^{+}$ bone marrow stromal cells (BMSCs), thereby promoting bone regeneration. Furthermore, genetic studies using Axin2-Cre; tdTomato, Inhba, and Wls conditional knockout mice underscored the essential roles of Wnt signaling in BMSCs and preNeu-derived activin A in orchestrating this regenerative process. The OdMac-regulated regenerative niche represents a promising therapeutic target for regenerative medicine.

**DECHIPERING THE ROLE OF P4HB IN OSTEOCLASTS AND IN IN VIVO BONE HOMEOSTASIS**

Luca Sandor, Edina Simon, Attila Mocsai, and David Gyori
*Department of Physiology, Semmelweis University, Budapest, Hungary*

Osteoclast are the unique bone-resorbing cells of the human body required for normal and pathological bone homeostasis. In our previous studies, we identified Prolyl 4-Hydroxylase Subunit Beta (P4HB) capable of regulating pre-osteoclast migration and osteoclast fusion within the solid tumor microenvironment during bone metastasis formation. To better understand the role of P4HB in osteoclasts and in skeletal diseases, we used transgenic mouse model. Previous studies of P4hb gene deletion demonstrated that P4HB is essential for cell viability, probably because of its critical function during protein folding. Thus, P4HB deletion in mice caused embryonic lethality. To overcome this problem, our experimental strategy was to generate P4HB conditional knockout animals. Using the Cre-loxP system to obtain lineage-specific deletion of P4HB, we generated P4hbΔOC transgenic mice (lacking the expression of P4HB in osteoclasts) by crossing Ctskcre/+ and P4hbflox/flox mice. P4hbΔOC animals were significantly smaller than wild-type (WT) littermates, had bent back and rounded face with serious limb abnormalities. Bone marrow-derived progenitors isolated from P4hbΔOCmice and differentiated into osteoclast in vitro in the presence of recombinant M-CSF and RANKL resulted in significantly decreased number and size of multinuclear polykarions compared to WT cultures. We are also in the process of performing micro-CT analysis of the long bones of WT and P4hbΔOC animals. Better understanding the role of P4HB in osteoclasts may facilitate the development of novel therapies for the treatment of skeletal diseases associated with pathological bone resorption, e.g., osteoporosis, autoimmune arthritis and cancer-related bone loss.

**DIRECT AND OSTEOBLAST-MEDIATED EFFECTS OF IL-1Β, TNF-Α AND IFNΓ ON HUMAN OSTEOCLASTOGENESIS**

Juliana Bousch, Lisa Rosenbaum, Christoph Suschek, and Uwe Maus

*Clinic for Orthopedics and Trauma Surgery, University Hospital Düsseldorf, Düsseldorf, Germany*

Proinflammatory cytokines are linked to inflammation-associated bone loss, but their effects are highly context-dependent. We previously found that IL-1β and TNF-α enhance mineralization in primary human osteoblasts (hOB). In the present study, we asked whether these pro-osteogenic effects coincide with pro- or anti-osteoclastogenic activity. We tested direct cytokine effects on osteoclastogenic differentiation of primary human CD14+ monocytes (hMON) and indirect effects mediated by the secretome of cytokine-treated hOB. hMON were differentiated for 23 days with RANKL/M-CSF and treated directly with IL-1β, TNF-α or IFNγ, or with conditioned medium (CM) from pooled hOB exposed to the same cytokines under osteogenic conditions (days 17–21). Osteoclastogenesis was quantified by TRAP activity (normalized to cell number) and counts of TRAP+ and TRAP− multinucleated cells (MNCs). hOB mineralization was assessed by Alizarin Red S. In hMON cultures, IFNγ stimulation markedly reduced multinucleation. Under TNF-α, multinucleated cells shifted toward a TRAP− phenotype, with no increase in TRAP+ MNCs. Following IL-1β exposure, multinucleation was strongly reduced. In the indirect co-cultures, CM from undifferentiated or differentiated hOB reduced MNC numbers of RANKL/M-CSF stimulated hMON. CM from IFNγ-treated hOB lowered TRAP activity but did not substantially alter MNC formation, whereas CM from TNF-α- or IL-1β-treated hOB markedly suppressed MNC formation. Overall, TNF-α promoted multinucleation without a TRAP+ osteoclast phenotype, consistent with macrophage-type giant-cell fusion rather than full osteoclast maturation. Moreover, CM from IL-1β- and TNF-α-primed hOB strongly suppressed RANKL/M-CSF-driven osteoclastogenesis in hMON. Together with enhanced hOB mineralization, this primary human cell model suggests a pro-osteogenic effect by IL-1β and TNF-α. This underscores context-dependent regulation of bone remodeling within an inflammatory bone microenvironment.

**DKK1 INHIBITION ALLEVIATES MITOCHONDRIAL DYSFUNCTION AND CELLULAR SENESCENCE VIA THE CGAS-STING-IFN-BETA-STAT3 PATHWAY IN AGED BONE**

Masayoshi Ishida[1], Rina Iwamoto[1], Takumi Takahashi[1], Zhifeng He[1], Nobuyuki Udagawa[2], and Yasuhiro Kobayashi[1]

[1]*Department of Hard Tissue Research, Institute for Oral Science, Matsumoto Dental University, Shiojiri, Japan*

[2]*Department of Biochemistry, Matsumoto Dental University, Shiojiri, Japan*

Background: Dickkopf-1 (Dkk1) is a potent Wnt signaling antagonist that inhibits osteoblast differentiation. While anti-Dkk1 neutralizing antibody treatment has been reported to restore bone mass in aged mice, the molecular mechanisms linking Dkk1 to cellular senescence and mitochondrial homeostasis remain poorly understood. This study aimed to elucidate whether Dkk1 is regulated by the cGAS-STING pathway and its contribution to the pro-senescent microenvironment in aged bone marrow. Methods: Aged C57BL/6 mice were treated with anti-Dkk1 neutralizing antibodies. Bone phenotypes were evaluated by mCT analysis, and cellular senescence was assessed using SPiDER-SA-b-gal staining along with p16/p21 expression. In vitro, the mesenchymal stem cell line ST2 was induced into senescence via replicative passage. We analyzed a panel of Senescence-Associated Secretory Phenotype (SASP) factors, including IL-6, IL-8, Cxcl10, CCL2, and IFN-b. To delineate the signaling axis, cells were stimulated with 2',3'-cGAMP or IFN-b, with or without anti-IFN-b neutralizing antibodies. Phosphorylation of STAT3 (p-STAT3) were assessed by immunoblotting. Mitochondrial integrity was evaluated using the JC-1 assay and quantification of cytosolic mtDNA leakage. Results: mCT analysis revealed that anti-Dkk1 treatment remarkably recovered bone mass in aged mice, with bone volume fraction (BV/TV) increasing from 4% to 8%. This recovery was accompanied by a significant reduction in SPiDER-SA-b-gal-positive senescent cells and SASP expression (IL-6, IL-8, Cxcl10, and CCL2) in bone tissue. In senescent ST2 cells, Dkk1 expression was markedly upregulated along with robust p-STAT3 enhancement. Dkk1 knockdown restored mitochondrial membrane potential and suppressed cytosolic mtDNA leakage. Crucially, we identified STAT3 binding sites within the Dkk1 promoter region. Treatment with the STAT3 inhibitor, Stattic, significantly downregulated Dkk1 expression, while anti-IFN-b neutralizing antibodies also abrogated Dkk1 induction. Furthermore, serum cGAMP levels in aged mice positively correlated with the frequency of Dkk1+ cells in the bone marrow. Conclusion: Our findings reveal a novel vicious cycle that accelerates cellular senescence, where age-related cGAS-STING activation drives Dkk1 expression via direct transcriptional regulation by the IFN-b-STAT3 axis. Dkk1, in turn, exacerbates mitochondrial dysfunction and mtDNA leakage, further fueling this cycle. Targeting the Dkk1- mediated loop represents a promising senomorphic strategy to restore mitochondrial integrity and bone formation in age-related bone diseases.

**DONOR MICROBIOTA AGE DELAYS FRACTURE PAIN RESOLUTION AND CALLUS MINERALIZATION IN FEMALE MICE**

Parker Kooima, and Joseph Roberts

*Nutritional Sciences, Arizona State University, Phoenix, United States*

Aging is associated with prolonged fracture pain and delayed bone repair, as well as distinct changes in gut microbiota before and after fracture. However, it remains unclear whether these microbiome changes are purely correlative consequences of injury and aging, or whether they actively contribute to post-fracture pain and bone repair. Our study aimed to determine if an aged microbiota, regardless of host age, would prolong pain sensitivity and delay fracture healing through altered inflammatory signaling. Young (12 wk) female C57BL/6J were randomized to receive fecal microbiota transplantation (FMT) from young (12 wk; young-FMT) or aged (18 mo; aged-FMT) donors prior to femur fracture. Pain behaviors (rearing, static weight bearing, thermal hyperalgesia) were assessed at baseline and days 3, 7, 14 and 21 post-fracture. Ipsilateral lumbar dorsal root ganglia (DRG) were isolated and used to measure gene expression and immune cell populations by qPCR and flow cytometry, respectively. Serum cytokines were quantified by multiplex ELISA. Fracture healing was assessed at day 21 using microCT. Young-FMT mice returned to baseline rearing behavior by day 7, whereas aged-FMT mice exhibited reduced rearing through day 21 post-fracture. Aged-FMT exhibited greater hindlimb weight-bearing asymmetry than young-FMT, with significant differences at days 3, 14 and 21. Relative to baseline, aged-FMT also displayed persistent thermal hyperalgesia through day 21 vs resolution in young-FMT by day 14. We next phenotyped the fracture callus to determine if colonization with an aged microbiota delays fracture healing and thus prolonging pain and functional deficits. Fracture healing was delayed in aged-FMT compared to young-FMT, indicated by a significantly decreased callus BV/TV and BMD. Phenotyping of lumbar DRG at day 7 post-fracture revealed a significant increase in gene expression of Ngf, Npy, Il10, and Ccl20 in aged-FMT mice, along with increased percentages of CD3+ T-cells but not CD11b+ myeloid cells. Serum MIP-1α and IL-10 were also increased in aged-FMT at day 7 post-fracture. Our results indicate that the composition of an aged microbiota, independent of host age, may worsen pain and delay fracture healing through inflammatory signals potentially involving T cell infiltration into DRG.

### EARLY HEMATOPOIESIS IN THORACIC AND ABDOMINAL AORTIC ANEURYSMS

Viola Niemann[1], Nicolas Stumpe[2], Niklas Gilleßen[1], Maren Döring[1], Alexander Brückner[3], Daniela Wenzel[3], Ulrich Flögel[2], and Maria Grandoch[1]

[1]*Institute of Translational Pharmacology, Heinrich-Heine-University, Düsseldorf, Germany*

[2]*Institute of Molecular Cardiology, Heinrich-Heine-University, Düsseldorf, Germany*

[3]*Life and Brain, University Bonn, Bonn, Germany*

Thoracic and abdominal aortic aneurysms (TAA/AAA) are life-threatening conditions that are treated only at advanced stages by surgery. Immune cell infiltration of the aortic wall drives extracellular matrix degradation and vascular remodeling during aneurysm development. In addition to circulating immune cells, hematopoietically active bone marrow (BM) represents a sustained source of immune cells contributing to disease progression. However, the precise timing of hematopoietic activation during aneurysm development remains unclear. Across preclinical cardiovascular models, e.g. myocardial infarction, dampening hematopoietic activation improves tissue remodelling, thereby providing a rationale to explore hematopoiesis as a therapeutic target in TAA/AAA. This study aimed to analyze early hematopoietic responses during AAA/TAA development. Male C57BL/6J mice (8–12 weeks) were treated with angiotensin II (AngII, 1000 ng/kg/min) and β-aminopropionitrile (BAPN, 0.1% in drinking water) to induce TAA and AAA. Aneurysms were assessed by magnetic resonance imaging, blood pressure (BP) measurements, histology, and flow cytometry of BM and aortic tissue. AngII/BAPN treatment induced TAA in 30%, AAA in 20%, and combined aneurysms in 42% of mice. Within one day of AngII exposure, BP increased and dilation of the thoracic aorta was detectable. At this early time point, initial immune activation was evident. Flow cytometry revealed a reduction of neutrophils and monocytes in the circulation and spleen, while histological analysis detected the first macrophages in the thoracic aortic wall. In contrast, alterations at the level of hematopoietic stem and progenitor cells in vertebral and femoral BM occurred only after four days. The concomitant decrease of progenitor cells and mature immune cells in the BM, together with increasing numbers of mature immune cells in the blood and in the aortic wall, indicates enhanced hematopoietic maturation and mobilization to sustain ongoing vascular inflammation. In conclusion, hematopoietic activation follows early hemodynamic and vascular immune changes and amplifies inflammatory processes during aneurysm progression. Targeting hematopoiesis may therefore represent a promising strategy to limit inflammatory vascular remodeling and disease progression.

**GENETICALLY MODIFIED HOXB8 PROGENITOR CELLS FOR FUNCTIONAL ANALYSIS OF OSTEOCLASTOGENESIS**

Dorottya Markó, Áron Pánczél, and Attila Mócsai

*Physiology, Semmelweis University, Budapest, Hungary*

Background: Osteoclasts are multinuclear giant cells originating from the myeloid lineage of the hematopoietic system. As the only cell type capable of bone resorption, they play a central role in maintaining skeletal homeostasis. Impaired osteoclast formation or function causes osteopetrosis, whereas excessive bone resorptive activity occurs during osteoporosis, inflammatory bone erosion, and osteolytic bone metastases. Due to their role in physiological and pathological bone metabolism, understanding the molecular and cellular mechanisms that control osteoclast biology remains a significant research priority. Methods: Our research group has previously established a HoxB8-based system in which mouse bone marrow cells were retrovirally transduced with ER-HoxB8 fusion protein. In the presence of β-estradiol, the HoxB8 is activated, resulting in the generation of conditionally immortalized HoxB8 progenitor cells. Upon β-estradiol withdrawal, these progenitors can be differentiated toward multiple myeloid cell types. Aims: Our aim is to develop a HoxB8 cell line that can be differentiated toward osteoclast-like cells and to apply the lentiviral CRISPR/Cas9 system for targeted gene deletions in these progenitors. Results: Upon stimulation with M-CSF and RANKL for six days, the HoxB8 progenitors differentiated toward multinucleated, TRAP-positive cells, confirming the osteoclastogenic potential of the system. We are currently performing CRISPR/Cas9-based gene editing to generate HoxB8 cell lines that lack RANK or DC-STAMP, supposedly leading to defective differentiation into osteoclasts and fusion into multinucleated cells, respectively. Conclusion: Our results demonstrate that the HoxB8 system is an efficient method for functional studies of osteoclast differentiation and activity.

**IMMUNE “TRAINING” DEMONSTRATED BY EXACERBATION OF INFLAMMATORY ARTHRITIS IS MEDIATED BY LYMPHOCYTES**

Claire Wingert

*Immunology and Microbial Pathogenesis, Weill Cornell Graduate School, Ithaca, United States*

Rheumatoid arthritis (RA) is an autoimmune disease mediated by a diverse array of immune cell subsets.Despite emerging new ideas, the specific pathologic mechanisms by which lymphocytes drive arthritis remain poorly understood. This project investigates the role of lymphocytes in the Zymosan-Induced Arthritis (ZIA) mouse model by leveraging both loss-of-function and gain-of-function perturbations to uncover how these cells promote synovitis across tissues. We first developed a model of recurrent inflammatory flares, identifying a marked exacerbation of both local and systemic inflammation in subsequent episodes. These flares are characterized by increased joint swelling, synovial tissue infiltration, alterations in bone structure, and elevated Serum Amyloid A (SAA) levels. Notably, mice deficient in lymphocytes failed to exhibit this exacerbation, indicating that the escalation of arthritic flares is dependent on the presence of adaptive immune cells. Furthermore, the administration of Immune Checkpoint Blockade (ICB) to activate lymphocytes significantly worsened ZIA pathology. This exacerbation was accompanied by increased neutrophil infiltration into the synovial tissue, elevated inflammatory cytokine production, and distinct shifts in hematopoietic stem and progenitor cell (HSPC) populations within the bone marrow. Collectively, these data confirm that lymphocytes orchestrate multi-tissue inflammation and play a critical role in driving the severity of recurrent arthritic flares.

**INFLAMMATORY MEDIATORS AND MIGRATORY POTENTIAL OF OSTEOCLAST PROGENITORS IN TYPE 1 DIABETES MELLITUS**

Danka Grcevic[1,2], Marta Radosevic[1,2], Masa Filipovic[1,2], Ozana Jaksic[1,2], Sara Anicic[1,2], Edi Rodak[3,4], Pavao Planinic[5], Natasa Kovacic[2,6], Tomislav Kelava[1,2], Sanja Novak[7], and Ivo Kalajzic[8]

[1]*Department of Physiology and Immunology, University of Zagreb School of Medicine, Zagreb, Croatia*

[2]*Croatian Institute for Brain Research, University of Zagreb School of Medicine, Zagreb, Croatia*

[3]*Department of Medicine, UConn Health, Farmington, United States*

[4]*Department of Histology and Embryology, University of Osijek Faculty of Medicine, Osijek, Croatia*

[5]*Department of Physiology, University of Mostar School of Medicine, Mostar, Bosnia and Herzegovina*

[6]*Department of Anatomy, University of Zagreb School of Medicine, Zagreb, Croatia*

[7]*Department of Physiology and Cell Biology, University of Arkansas for Medical Sciences, Little Rock, United States*

[8]*Center for Regenerative Medicine and Skeletal Development, UConn Health, Farmington, United States*

Type 1 diabetes (T1D) is an autoimmune disease characterized by destruction of insulin-producing beta-cells, metabolic changes, chronic inflammation, and systemic adverse effects. While T1D-associated osteopathy was previously observed, the cellular and molecular mechanisms that accelerate osteoclast-mediated bone loss remain unclear. Therefore, we aimed to assess phenotype and migratory potential of osteoclast progenitors (OCPs) in a mouse model of streptozotocin(STZ)-induced diabetes and human samples of T1D. OCPs from human peripheral blood and STZ-injected mice were phenotyped and subjected to in vitro migration assays. In vivo migration of CX3CR1+ OCPs was evaluated using CX3CR1/GFP reporter knock-in/knock-out mice, and lineage tracing was performed using inducible CX3CR1CreERT2/Ai9(TdTomato) mice. We identified mouse (CD45+CD3−B220−NK1.1−Ly6G−CD11blow/+CD115+) and human (CD45+CD3−CD19−CD56−CD15−CD11b+CD14+) OCPs in T1D, with the additional expression of activation markers (RANK, CCR2, CD64, TLR4, CD27). T1D-derived OCPs exhibited enhanced migration in vitro toward CCL2 gradient. Bone sections of STZ-treated homozygous CX3CR1/GFP mice (lacking CX3CR1 surface expression) had fewer GFP+ cells than heterozygous CX3CR1/GFP (expressing CX3CR1) littermates. Parabiosis between heterozygous CX3CR1/GFP control and wild-type T1D mouse confirmed the migration of GFP+ cells, detected on the bone sections of wild-type counterpart. Lineage tracing of CX3CR1+ TdTomato-labeled OCPs confirmed their migration to bone surfaces and fusion into multinucleated TRAP+ osteoclasts. Our study showed that circulatory OCPs increasingly express activation receptors and may contribute to bone resorption in T1D.
This work was supported by the European Union through National Recovery and Resilience Plan under grant agreement NPOO.C3.2.R3-I1.04.0093 (CHIPART); project No. 10106-25-3013 (MEDIS); project No. 10106-25-2987 (LIOS); Croatian Science Foundation HRZZ-IP-2022-10-2285 (OPTIMIDAL).

**INVESTIGATING THE ROLE OF BISPHOSPHONATES ON IMMUNE CELLS FOR THERAPEUTIC DEVELOPMENT FOR OSTEONECROSIS OF THE JAW (ONJ)**

Claudia Wang[1], Lauren Surface[2], Dylan Kuennen[2], and Nita Singh[2]

[1]*Cellular and Molecular Biology, University of Michigan, Ann Arbor, United States*

[2]*Biologic & Materials Sciences and Prosthodontics, University of Michigan, Ann Arbor, United States*

Nitrogen-containing bisphosphonates (BPs) are a class of bone-targeting drugs that reduce bone reabsorption by inhibiting osteoclast differentiation and function. While these are widely prescribed drugs for both bone metastases and osteoporosis, patients harbor a significant risk of developing osteonecrosis of the jaw (ONJ), a rare but painful condition where the exposed jawbone fails to heal after oral trauma. The underlying mechanisms of how BP treatment results in ONJ remain poorly understood. Recent studies suggest that BPs enhances pro- inflammation responses following oral perturbation, leading to ONJ development. Specifically, circulating, free BPs are internalized by immune cells outside of the skeleton, in which their function and cellular identity may consequently be altered to induce chronic inflammation. This project aims to develop therapeutic targets for preventing ONJ development by identifying immune cells that may uptake BPs in the jaw. To do so, I injected fluorescently labeled zoledronate (5FAM-ZOL) or 5FAM retro-orbitally into young adult mice and, 24 hours later, sorted cells by flow cytometry to perform single-cell RNA-sequencing. Our preliminary data suggests that a subpopulation of immune cell types, including pro-inflammatory macrophages, internalize 5FAM-ZOL in the jaw, which may contribute to the altered immune status observed in ONJ. Given this preliminary finding, I hypothesize that BPs contribute to chronic inflammation in ONJ by directly promoting pro-inflammatory macrophages phenotypes and signaling. To test this hypothesis, I will use our previously established ONJ mouse model to observe BP-internalizing macrophages polarize towards more pro-inflammatory than anti-inflammatory phenotype over ONJ disease progression. I will also employ this model and administer a therapeutic approach to suppress BP-induced macrophage inflammatory responses for proper healing to prevent ONJ development.

**LOSS OF TFR2 UNLEASHES NEUTROPHIL-LIKE INFLAMMATORY PROGRAMMING IN OSTEOCLAST PROGENITORS AND DRIVES INFLAMMATORY BONE LOSS**

Agnus Davis, Maria Ledesma-Colunga, Michael Frech, Sofia Hutter, Thea Hofmann, Annika Häuser, Susnigda Sen, Cornelia Stoll, Alexandra Correa Zamora, Lorenz Hofbauer, Georg Schett, Ulrike Baschant, and Carina Scholtysek

*Medizin 3: Rheumatology und Immunology, Uniklinikum Erlangen, Erlangen, Germany*

Iron homeostasis is a critical regulator of bone remodelling, with both iron deficiency and overload disrupting the balance between osteoclast and osteoblast activity. While transferrin receptor 1 (TFR1) is well established as the primary mediator of cellular iron uptake, transferrin receptor 2 (TFR2) functions as a systemic iron sensor and has been predominantly studied in hepatocytes. However, the role of TFR2 in osteoclast biology and inflammatory bone loss remains poorly understood. Here, we investigate the role of TFR2 in osteoclasts using inflammatory bone loss models and in vitro approaches. Under steady-state conditions, wild-type and Tfr2-deficient mice displayed comparable bone mass. In contrast, inflammatory challenges, including experimental arthritis and periodontitis, revealed a pronounced phenotype in Tfr2 knockout mice, characterized by enhanced alveolar bone loss, elevated osteoclast numbers, and increased joint destruction. These findings indicate a context-dependent protective role for TFR2 during inflammation-driven bone resorption. Mechanistically, Tfr2-deficient bone marrow–derived osteoclasts exhibited increased fusion and resorptive activity in vitro. Metabolic profiling uncovered elevated oxidative phosphorylation and glycolytic activity in Tfr2-deficient osteoclasts, which was further enhanced under inflammatory conditions. Notably, quantitative iron measurements demonstrated preferential mitochondrial iron accumulation without substantial alterations in cytosolic iron levels, suggesting dysregulated intracellular iron partitioning rather than global iron overload. Transcriptomic analysis identified altered expression of genes associated with oxidative stress responses and mitochondrial quality control pathways. Collectively, these data identify TFR2 as a previously unrecognized regulator of osteoclast metabolism and iron handling, acting as a checkpoint that restrains osteoclast activity under inflammatory conditions. These findings highlight a novel link between iron sensing, mitochondrial metabolism, and osteoimmune-driven bone loss, with potential implications for inflammatory bone diseases.

**OSTEOSARCOMA HIJACKS LOCAL BONE MARROW HEMATOPOIESIS TOWARD MYELOID CELL EXPANSION AND ATF3 ACTIVATION**

Irene Fischetti[1], Chiara Ratti[1], Valeria Cancila[2], Claudio Tripodo[3], Cristina Meazza[4], and Claudia Chiodoni[1]

[1]*Experimental Oncology, Fondazione IRCCS Istituto Nazionale dei Tumori, Milan, Italia*

[2]*Tumor Immunology Unit, Department of Health Sciences, University of Palermo, Palermo, Italia*

[3]*Advanced Pathology Laboratory, IFOM ETS-The AIRC Institute of Molecular Oncology, Milan, Italia*

[4]*Pediatric Oncology, Fondazione IRCCS Istituto Nazione dei Tumori, Milan, Italia*

Osteosarcoma (OS) is the most common primary bone cancer in children and adolescents. Despite the introduction of new treatment options (still based mainly on multimodal chemotherapies and surgery), the 5-year overall survival rate for osteosarcoma has remained stagnant for several decades, ranging from 60–70% for localized cases and only 20–30% for metastatic cases. In recent years, it has become increasingly clear that the immune microenvironment plays a critical role in determining OS aggressiveness and resistance to therapies. A deeper understanding of OS interactions with the surrounding bone marrow (immune) microenvironment, is essential for refining existing therapies and developing novel, more effective treatments. We have recently demonstrated, in a different context (i.e., breast cancer), that the bone marrow represents an early sensor of an incipient distant neoplasia and responds to such hint with significant transcriptional reprogramming and stromal changes. We identified mesenchymal stem cells as the very first sensors, which in turn, by the release of IL-1b, induce the upregulation and nuclear translocation of the transcription factor ATF3 in hematopoietic stem cells, driving myeloid cell differentiation and expansion that further sustain tumor progression. Considering that the bone represents the local microenvironment in which OS develops and progresses, we hypothesized that a similar mechanism could take place also in the case of OS.To test our idea, we injected mouse OS cells into the tibial bone of syngeneic animals and analyzed myeloid cell markers, as well as the expression of AFT3 in residual bone marrow areas adjacent to tumor areas. MPO staining, as marker for myeloid elements, showed an overall increase in the number of MPO+ cells and a re-distribution of positive cells in myeloid clusters in specific areas. We also detected increased positivity for ATF3 expression and its nuclear localization in such areas when compared with naïve bone marrow samples.Although preliminary, our data suggest that OS cells induce a reprogramming of the neighboring bone marrow, leading to myeloid pool expansion and ATF3 activation. We will now examine whether this expansion and the trancripton factor ATF3 have any functional role in OS growth and metastatic dissemination.

### SIX HOUR DECALCIFICATION: A MICROWAVE ENHANCED WORKFLOW FOR 3D IMAGING OF MINERALIZED TISSUES

Eric Greto, and Daniela Weidner

*Medical Clinic 3, University Hospital Erlangen, Erlangen, Germany*

Three-dimensional imaging of mineralized tissues, such as bone and adjacent structures like the synovium, requires effective decalcification to enable deep optical penetration for light-sheet or two-photon microscopy. EDTA-based decalcification remains the gold standard because it preserves antigenicity, but typically requires 7–10 days, creating a major bottleneck in imaging workflows. Microwave-assisted decalcification has been shown to accelerate mineral removal, yet conventional microwave protocols risk overheating and damaging soft tissues needed for antibody or RNA labeling. Commercial laboratory microwave systems such as the Pelco BioWave improve reagent penetration and are advertised to decalcify bone within 24 hours, though in our hands complete decalcification of a mouse femur required approximately 48 hours. To overcome these limitations, we redesigned the hardware and workflow to improve thermal control and microwave exposure efficiency. The standard BioWave setup uses a large water volume for cooling, which slows heating and cooling cycles. We developed a custom 3D-printed carrier that positions the sample inside a glass tube surrounded by a narrow 3–4 mm water jacket, reducing thermal mass and keeping the bone within ~1 cm of the tube surface. This geometry increases microwave energy delivery while enabling rapid active cooling. Using this optimized system, we achieved a substantial acceleration of EDTA-based decalcification. Mouse femurs became radiolucent within 4 hours—comparable to 7-day room-temperature decalcification—and whole mouse legs reached sufficient softness for synovial imaging within 6 hours. Importantly, we observed no detectable loss of antigenicity. These results demonstrate that controlled microwave-assisted decalcification, enabled by improved sample geometry, can dramatically shorten preparation times while preserving tissue integrity, offering a powerful enhancement for high-resolution 3D osteological imaging.

### SPLICING FACTOR SRSF1 CONTROLS SYSTEMIC INFLAMMATION METABOLISM AND FUNCTION OF MYELOID DERIVED SUPPRESSOR CELLS

Hao-Yun Peng[1], Maxime Adrien Mohamed Jeljeli[1], Darleen Hueser[2], Trang Luong[1], Hiroki Furuya[1], Tsian Zhang[1], Alexander Marleev[3], Alina Marusina[3], Jingyi Wang[1], Emanual Maverakis[3], Towia Libermann[4], John Asara[5], Anika Grueneboom[2], and Iannis E. Adamopoulos [1]

[1]*Department of Rheumatology, Beth Israel Deaconess Medical Center / Harvard Medical School, Boston, United States*

[2]*Biospectroscopy / Bioimaging, Leibniz-Institut für Analytische Wissenschaften – ISAS – e.V., Dortmund, Germany*

[3]*Department of Dermatology, University of California, Sacramento, United States*

[4]*Genomics, Proteomics, Bioinformatics and Systems Biology Center, Beth Israel Deaconess Medical Center / Harvard Medical School, Boston, United States*

[5]*Division of Signal Transduction/Mass Spectrometry Core, Beth Israel Deaconess Medical Center / Harvard Medical School, Boston, United States*

Immune suppression following immune activation is a critical event in ensuring homeostasis and its imbalance leads to chronic inflammation as typically occurs in autoimmune diseases. Although IL-17 signaling has been extensively studied in the context of immune activation, its role in suppression of inflammation has been overlooked and remains elusive. Herein, we show that Serine/arginine-rich splicing factor 1 (SRSF1), an essential RNA-binding protein that controls post-transcriptional gene expression downstream of the IL-17 receptor complex regulates the frequency and the immune responses of Ly6G+ myeloid-derived suppressive cells (MDSC) during IL-17A-mediated inflammation. Specifically, genetic ablation of Srsf1 in myeloid cells results in the downregulation of the de novo lipid biosynthesis and the impairment of the suppressive function of the granulocytic subset of myeloid-derived suppressive cells in vitro and in vivoin two independent animal models of disease. Moreover, RNAseq, and lipidomic analysis revealed palmitic acid biosynthesis as a key pathway in maintaining functional MDSC's. Our data uncovers a novel role of SRSF1 in the control of myeloid cell homeostasis and function downstream of IL-17 receptor and provide insights into the pathogenesis of chronic inflammatory disorders.

**TARGETED ACTIVATION OF STAT3 IN BONE ENHANCES FRACTURE HEALING**

Jiliang Li, and Mariana Moraes de Lima Perini

*Biology, Indiana University, Indianapolis, United States*

Signal Transducer and Activator of Transcription 3 (Stat3) is a key regulator of bone homeostasis and has been implicated in both osteogenesis and angiogenesis. However, the therapeutic potential of Stat3 activation specifically within osteoblasts during fracture repair remains poorly defined. We hypothesized that selective activation of Stat3 in osteoblasts enhances bone formation and accelerates fracture healing.To test this hypothesis, we generated a doxycycline-inducible binary transgenic mouse model that overexpresses constitutively active Stat3 (Stat3C) selectively in the osteoblast lineage (OB-Stat3C mice). Using this model, we have demonstrated that the activation of Stat3 in osteoblasts lead to significantly greater amount of lamellar bone formation compared to the control mice. Due to the known role of Stat3 in oncogenesis, long-term skeletal tumor formation was assessed up to 18 months of age.Mice received doxycycline from birth until three weeks prior to fracture surgery to prevent excessive marrow cavity mineralization. At 16 weeks of age, stabilized transverse femoral fractures were created in OB-Stat3C mice and littermate controls. Fracture healing was monitored for four weeks post-injury using biweekly radiographic imaging. Micro-computed tomography (micro-CT) was performed to quantify callus bone volume fraction (BV/TV) and trabecular microarchitecture.Radiographic imaging revealed earlier callus formation in OB-Stat3C mice compared with control mice, indicating accelerated fracture repair. Micro-CT analysis demonstrated a significant increase in callus BV/TV by 29.32% in male mice and 17.52% in female mice, as well as increased trabecular number by 22.69% in males and 12.67% in females. Importantly, no evidence of skeletal tumor formation was observed in OB-Stat3C mice up to 18 months of age, supporting the safety of osteoblast-specific Stat3 activation.These findings demonstrate that targeted activation of Stat3 in osteoblasts enhances both the timing and quality of fracture callus formation. Given the established role of Stat3 in osteogenic and angiogenic signaling, the accelerated healing response likely reflects coordinated effects on osteogenesis and vascularization. Ongoing studies are focused on defining the contribution of Stat3-mediated angiogenesis during fracture repair.This study provides compelling evidence that osteoblast-specific Stat3 activation is a safe and effective strategy to enhance bone formation and accelerate fracture healing. Targeting Stat3 signaling in bone represents a promising therapeutic approach for improving skeletal repair following injury.

**THE IMPACT OF CHRONIC COLITIS ON BONE MARROW NICHE STRUCTURE**

Calar Sedik Jafar, Benedikt Meier, Yanina Ostendorf, and Maria Grandoch

*Institute of Translational Pharmacology, Medical Faculty and University Hospital Düsseldorf, Heinrich Heine University Düsseldorf, Düsseldorf, Germany*

Inflammatory bowel diseases (IBD), such as Crohn’s disease and ulcerative colitis, are characterized by relapsing intestinal inflammation. Besides other potential risk factors, a dysregulated immune response is considered a major cause. The bone marrow (BM) niche, containing hematopoietic and mesenchymal stem cells (HSC, MSC), provides a specialized environment essential for hematopoiesis. However, it is yet unknown whether and how the BM niche remodels in chronic colitis, thus impacting the inflammatory response. We hypothesize that repeated DSS-induced inflammation remodels the BM niche in a site- and time-dependent manner, altering vascular structure and influencing HSC trafficking and lineage decisions. Male 5 to 8 week old C57BL/6J and apolipoprotein E deficient mice received one to five cycles of a dextran sodium sulfate (DSS, 2%) treatment followed by 2-week washouts. HSC and mature leukocyte populations were quantified by flow cytometry in femur, tibia, sternum, and vertebrae. BM sections were stained for endomucin to quantify sinusoids and for hyaluronic acid (HA) to assess matrix composition. The trabecular bone (TB) fraction was analyzed on hematoxylin stained sections. Chronic colitis was confirmed by assessing body-weight loss, an increased colon weight-to-length ratio, and histological evidence of inflammation in the colon. After three DSS cycles, flow cytometry showed expansion of stem and progenitor cells with myeloid skewing, most pronounced in the femur. In the femoral metaphysis, sinusoidal density increased after three cycles, suggesting vascular remodeling. HA staining in the BM remained unchanged. The bone structure analysis indicated a reduced TB fraction in the treated mice. Our findings indicate that repetitive cycles of experimental colitis remodel the BM niche, marked by increased numbers of sinusoids and TB loss, changes that may facilitate cell egress from the BM and potentially increase the available niche space for hematopoiesis.

**THREE-DIMENSIONAL VISUALIZATION OF COMPLETE VASCULAR NETWORK AND NEUTROPHIL CLUSTERS IN MURINE KNEE JOINTS DURING INFLAMMATORY ARTHRITIS**

Malte Roessing[1], Davide Panzeri[1], Darleen Hueser[1], Lara Janz[1], Leon Ben Achour[1], Rico Zapf[2], Flora Weber[1], Martin Stenzel[1], Oliver Aust[1], Thorsten Reichert[3], Ulrike Steffen[2], Matthias Gunzer[4], Jianxu Chen[1], and Anika Grueneboom[1]

[1]*Biospectroscopy, Leibniz Institut für Analytische Wissenschaften- ISAS - e.V, Dortmund, Germany*

[2]*Department of Internal Medicine 3, Friedrich-Alexander-University Erlangen-Nürnberg and Universitätsklinikum Erlangen, Erlangen, Germany*

[3]*Department of Cranio-Maxillo-Facial Surgery, University of Regensburg, Regensburg, Germany*

[4]*Institute for Experimental Immunology and Imaging, University Hospital, University Duisburg-Essen, Essen, Germany*

Rheumatoid arthritis (RA) is characterized by chronic inflammation of synovial joints, vascular remodeling, and immune cell infiltration. While inflammatory-mediated angiogenesis is a pivotal factor in joint destruction, the three-dimensional vascular architecture, its remodeling, and the associated impact on immune cell infiltration remain inadequately understood. This is mainly due to technical challenges in optical visualization and analysis of vascular networks within highly autofluorescent and scattering joint tissues. In this study, we investigated the vascular network and neutrophil distribution in murine knee joints under physiological conditions and in the collagen-induced arthritis model using optical tissue clearing and light-sheet fluorescence microscopy. Although optical tissue clearing aligns refractive index (RI) mismatches in tissues, challenges like autofluorescence, residual absorbing structures, and minor RI mismatches impair optical penetration depth. To address these limitations, we developed a motorized rotational bio-imaging tool for the Miltenyi UltraMicroscope Blaze that enables acquisition of complementary images from multiple angles and computational multi-view fusion, resulting in enhanced structural resolution across the sample volume. Using this multi-view strategy, we visualized the complete 3D vascular network of murine knee joints for the first time. This enabled quantitative analyses of vessel size, branching, and density, along with spatial mapping of neutrophil clusters in the joint in health and disease. Our results demonstrate that vascular remodeling is temporally and spatially associated with neutrophil infiltration, providing insight into inflammation and vascular remodeling during RA progression. This approach offers a high-resolution platform for 3D imaging of intact joints and other tissues, enabling integrated structural and cellular analyses of inflammatory processes.

**TIME-DEPENDENT CHANGES IN THE IMMUNE AND SKELETAL SYSTEMS FOLLOWING IRRADIATION AND HEMATOPOIETIC STEM CELL TRANSPLANTATION**

Priti Gupta[1], Riddhi Vyas[1], Nour Dada[1], Mattias Svensson[1], Marie Lagerquist[2], Carmen Corciulo[3], Tibor Sághy[1], and Cecilia Engdahl[1,2]

[1]*Dep. of Rheumatology and Inflammation, University of Gothenburg, Gothenburg, Sweden*

[2]*Dep. of Internal Medicine, CBAR, University of Gothenburg, Gothenburg, Sweden*

[3]*Department of Pharmacology, University of Gothenburg, Gothenburg, Sweden*

Hematopoietic stem cell transplantation (HSCT) following irradiation is a critical therapy for hematologic malignancies and immune disorders; however, the long-term effects on immune and bone health remain incompletely understood. In particular, the effects of irradiation and HSCT on susceptibility to inflammatory diseases, and their combined impact on bone loss, remain unclear. In this study, we investigated immune and skeletal responses in a mouse model where local antigen-induced arthritis (AIA) was induced either early (2 weeks) or late (10 weeks) after irradiation and HSCT. Irradiation and HSCT did not prevent the development of local arthritis, as joint swelling and synovitis were similar in both irradiated-HSCT and non-irradiated control mice at each time point. However, late induction of arthritis was linked to greater bone and cartilage damage in irradiated-HSCT mice compared to controls. Skeletal analyses show that irradiation and HSCT significantly reduced trabecular bone mineral density and cortical thickness in both arthritic and non-arthritic sides. Although arthritis caused additional local trabecular bone loss, the extent of arthritis-related bone loss was not further worsened by irradiation or HSCT. Consistent with these findings, serum CTX-I levels increased after irradiation and HSCT, indicating increased systemic bone resorption. Immune profiling revealed significant, time-dependent changes across stages following irradiation and HSCT, compared with controls. There was an early rise in granulocytes and monocytes, followed by a delayed recovery of lymphocytes, along with increased serum TGF-β1 levels compared to non-irradiated control mice. Humoral immune responses were specifically impacted. Antigen-specific IgG responses decreased at later time points after irradiation and HSCT, even though overall IgG levels remained stable. This suggests impairment of both innate and adaptive immunity at different stages following irradiation and HSCT. Finally, irradiated-HSCT mice exhibited persistent systemic effects, including reduced body weight, thymic and uterine atrophy, increased bone marrow apoptosis, and marrow adiposity, indicating prolonged post-transplant stress compared to non-irradiated controls. Overall, these findings indicate that irradiation and HSCT induce long-lasting, time-dependent changes in the immune system and bones. These factors affect inflammatory responses and increase bone fragility after irradiation and HSCT. Our results highlight critical periods of vulnerability after transplant with important implications for managing inflammatory and skeletal problems in HSCT survivors.

**TIME-RESTRICTED FEEDING ENGAGES THE GUT-IMMUNE-BONE AXIS TO ATTENUATE AGE-RELATED BONE LOSS**

Joseph Roberts, Michael Jakiche, Graham Collis, and Kelley Chau

*College of Health Solutions, Arizona State University, Phoenix, United States*

Aging is accompanied by progressive deterioration of bone microarchitecture, driven in part by dysregulated osteoclast activity and chronic inflammation. An popular geroprotective and anti-inflammatory dietary approach to maintaining health is called time-restricted feeding (TRF) that involves aligning food consumption to the body's circadian rhythm by consuming food during a short window of time. However, the impact of TRF on bone health remains unclear. The objective of our study was to address this knowledge gap by examining the impact of long-term adherence to TRF on bone microstructure. Female C57BL/6J mice (10 weeks of age) were randomly assigned to either ad libitum food access or time-restricted feeding (TRF) for 9 weeks, with TRF mice granted access to food for 8 hours during the dark cycle. Compared to baseline 10-week-old mice, prolonged TRF significantly attenuated age-related declines in trabecular bone microstructure within the femur and tibia without changing body weight or energy intake. Mechanistically, TRF reduced osteoclast formation, TRAP activity, and expression of key osteoclastogenic genes, and shifted the systemic inflammatory milieu toward an anti-inflammatory profile characterized by reduced proinflammatory cytokines IL-17A and increased IL-10, which where negatively and positively correlated with trabecular bone outcomes, respectively. TRF also induced tissue-specific regulation of the B-cell chemokine Cxcl13, with decreased expression in the small intestine and increased expression in bone marrow, accompanied by elevated serum CXCL13 levels. Disruption of the gut microbiota using a broad-spectrum antibiotic cocktail significantly reduced serum CXCL13, implicating microbial regulation of TRF-induced chemokine signaling. Consistent with this, microbiome profiling revealed that TRF significantly remodeled gut microbiota community structure, including enrichment of taxa associated with metabolic health and healthy aging. Together, these findings identify TRF as a simple dietary pattern that preserves skeletal microstructure, potentially through coordinated effects on osteoclast biology, immune signaling, and the gut-bone axis.

### Sponsors

Aegean Conferences and the conference organizers wish to gratefully acknowledge the following sponsors for their generous support of this meeting.

THE JAPANESE SOCIETY FOR BONE AND MINERAL RESEARCH

JSBMR

SINCE 1967

ASBMR™

THE AMERICAN SOCIETY FOR BONE & MINERAL RESEARCH

REGENERON
